# Stroke and motor outcomes are associated with regional and age‐specific changes in periodic and aperiodic cortical activity

**DOI:** 10.1113/EP093171

**Published:** 2025-09-24

**Authors:** Asher J. Albertson, Eric C. Landsness, Margaret Eisfelder, Brittany M. Young, Bradley Judge, Matthew R. Brier, Matthew J. Euler, Steven C. Cramer, Jin‐Moo Lee, Keith R. Lohse

**Affiliations:** ^1^ Department of Neurology Washington University School of Medicine St Louis Missouri USA; ^2^ Department of Neurology University of California Los Angeles, and California Rehabilitation Institute Los Angeles California USA; ^3^ Department of Psychology University of Utah Salt Lake City Utah USA; ^4^ Program in Physical Therapy Washington University School of Medicine St Louis Missouri USA

**Keywords:** aging, cerebral cortex, electroencephalogram, excitability, magnetic resonance imaging, stroke

## Abstract

Historically, stroke and ageing have been associated with changes in narrow‐band periodic neuronal activity, but recent work has highlighted the importance of broad‐band aperiodic activity. Aperiodic activity is represented by the 1/*f* slope of power spectral density generated by cortical activity. Here we explored changes in both periodic and aperiodic cortical activity in neurologically intact individuals and individuals with stroke, across the lifespan. We compared ‘resting state’ electroencephalograms from all participants after applying the *specparam* algorithm, which decomposes the power spectrum into aperiodic and periodic components. We also correlated motor outcomes to average whole cortex spectral slopes within the stroke group. We found a significant flattening (decrease in exponent) of power spectral slope with normal ageing. We found that both ageing and stroke were associated with fewer periodic peaks. Interestingly, we found that stroke was associated with a significant increase in spectral slope, but age moderated this effect. Younger stroke patients showed minimal difference in slope while older stroke patients had significantly steeper slopes (opposite to the direction in normal ageing). We next investigated the lesion locations most associated with changes in slope. Deep lesions were observed to have the greatest influence on cortical spectral slope. Finally, the slope in the stroke group was associated with performance on a test of manual dexterity, but this association was stronger in older individuals, and varied by scalp region. Our data suggest that stroke in the aged brain has unique effects on aperiodic activity possibly reflecting unique influence of injury on cerebral excitation/inhibition balance in aged individuals.

## Abstract (highlights)

- **What is the central question of this study?**
  What are the age‐specific effects of stroke on periodic and aperiodic cortical activity, and do these patterns relate to motor outcomes?
- **What is the main finding and its importance?**
  Stroke disproportionately steepens aperiodic spectral slope in aged individuals, particularly with deep lesions, and this change correlates with worse motor performance, suggesting it may serve as a biomarker of poor recovery in older stroke patients.

## 1 INTRODUCTION

Age is the largest non‐modifiable risk factor for stroke (Boehme et al., 2017; Chen et al., 2010; Dufouil et al., 2017; Rothwell et al., 2005). Ischaemic stroke survival is markedly reduced in aged individuals, and long‐term disability is significantly increased (Marini et al., 2004). Ischaemic stroke is also associated with an increased risk of dementia (Guo et al., 2018; Koton et al., 2022), with age being a significant risk factor for combined death and dementia (Allan et al., 2011) after stroke. In contrast, older age is associated with lower risks of post‐stroke epilepsy (Graham et al., 2013) and depression (Shi et al., 2017). The drivers of age‐related differences in post‐stroke cerebral function are poorly understood. Brain weight (Terry et al., 1987), myelinated fibres (Marner et al., 2003) and cortical thickness (Salat et al., 2004) all decline with age and are accompanied by changes in synaptic dynamics (Mostany et al., 2013), neuron structure (Dickstein et al., 2013), synaptic plasticity (Ou et al., 1997; Shankar et al., 1998) and network functional connectivity (Albertson et al., 2022; Grady et al., 2016; Wu et al., 2011). Stroke also causes short‐ and long‐term changes in brain structure (Brodtmann et al., 2020; Sayed et al., 2020), synaptic function (Brown et al., 2007; Hagemann et al., 1998; Lohse et al., 2025) and network connectivity (Bauer et al., 2014; Hakon et al., 2018; Siegel et al., 2016). The differential effect of stroke on brain structure and function in aged individuals is less well understood.

Periodic spontaneous neuronal activity (defined as rhythmic neuronal oscillations within canonical frequency bands) changes substantially with age and correlates with cognitive performance (Klimesch, 1999; Vlahou et al., 2014). Stroke also causes significant changes in spontaneous neuronal activity. Hyper‐acute stroke is associated with a broadband decrease in cortical spectral power (Kamitaki et al., 2019). Dynamic increases in low frequency power have been reported at various time points after stroke (Giaquinto et al., 1994; Laaksonen et al., 2013; Lu et al., 2001). Interestingly, increased low frequency activity after stroke is associated with both larger injury size and greater motor recovery (Cassidy et al., 2020). Whether stroke uniquely affects spontaneous periodic activity in aged vs. young individuals is incompletely characterized.

While changes in periodic cortical activity in ageing and stroke have received significant attention, a separate body of work has demonstrated the independent importance of aperiodic activity in cerebral function (Donoghue et al., 2020; He et al., 2010; Podvalny et al., 2015; Voytek et al., 2015). Aperiodic activity is represented by the 1/*f* slope of power spectral density generated by cortical activity. Cortical field potentials measured by electroencephalography (EEG) exhibit a characteristic negative slope, with the greatest power at lower frequencies (Donoghue et al., 2020). Disruptions in 1/*f* spectral slope have been identified in neuropsychiatric disorders including schizophrenia (Peterson et al., 2023) and attention deficit hyperactivity disorder (Ostlund et al., 2021). Simulation and in vivo data have suggested that 1/*f* slope inversely reflects excitation–inhibition balance (Gao et al., 2017). Similar to periodic activity, there are significant changes in aperiodic activity with age, and these changes correlate to performance on cognitive tests (Pathania et al., 2022; Voytek et al., 2015). Recent work has demonstrated that spectral slowing after stroke is a result of both periodic (diminished high frequency oscillations) and aperiodic (increased spectral slope) (Johnston et al., 2023) changes.

Given the relevance of neuronal activity (Vahdat et al., 2021), synaptic function (Michiels et al., 2023) and, importantly, disrupted excitation–inhibition balance (Boddington & Reynolds, 2017) to ischaemic brain injury and recovery, we approached the overarching question of whether stroke uniquely influences periodic and aperiodic activity in aged and young brains. We compared resting state EEGs from both healthy young and aged individuals to young and aged individuals with stroke. We applied the *specparam* algorithm (formerly *fitting oscillations with one‐over‐f*, or *FOOOF*) (Donoghue et al., 2020) to each EEG time series at each electrode to quantify the aperiodic (1/*f* slope) activity and periodic activity (power in narrowband peaks) for each participant, as previously described (Euler et al., 2024). Within the periodic data, we compared the total number of narrowband peaks, the central frequency of each peak and power at that frequency across the age range. Within the aperiodic data, we compared exponent and offset across the age range. Our aims were three‐fold: first, to examine the effect of stroke on the power spectrum across the age range; second, to explore how lesion location affects the power spectrum in the subset of participants with stroke; and finally, to examine the relationship between aperiodic activity and functional outcome across the observed age range after stroke. In all analysis, we model age as a continuous variable but in some figures, we show categorical age groups for ease of interpretation.

## 2 METHODS

### 2.1 Ethics statement

All analyses were performed using data from previously published work (Table 1). Original data collection was conducted in accordance with the protocol approved by the respective institutions’ review boards (as described). The secondary analysis described here was approved by the institutional review board at Washington University.

**TABLE 1:** Demographic statistics and spectral parameterization fits for each sample.

	Unimpaired Controls			
Variable	Babayan et al.	Euler et al. (combined)	Pathania et al.	Stroke Individuals Cassidy et al.
Participant characteristics				
No. of participants	114	82	38	61
Age, median [min, max] (years)	28 [23, 78]	22 [18, 46]	65 [19, 83]	59 [23, 86]
Gender (*n* (%)) Men Women Other	74 (65%) 40 (35%) 0 (0%)	30 (37%) 51 (62%) 1 (1%)	12 (32%) 26 (68%) 0 (0%)	41 (67%) 20 (33%) 0 (0%)
Days from stroke to EEG, median [min, max]	NA	NA	NA	106 [3, 3435]
Lesioned hemisphere left (*n* (%))	NA	NA	NA	27 (44%)
Dom. hemisphere affected? Yes (*n* (%))	NA	NA	NA	33 (54%)
Lesion volume, median [min, max] (ml)	NA	NA	NA	5.44 [0.03, 100.61]
EEG characteristics				
Hardware system	actiCAP active electrode system, BrainAmp MR, BrainProducts	WaveGuard Cap, Advanced Neurotechnology, ANT Company	actiCAP active electrode system, BrainAmp DC, BrainProducts	HydroCel Geodesic Sensor Net, magstim egi
No. of original channels and montage	62 Channels, 10‐10	64 Channels, 10‐10	32 Channels, 10‐10	256 Channels, Hydrocel GSN
No. of complete harmonized channels (out of 28), median [IQR]	27 [26, 28]	25 [23, 26]	24 [21, 26]	25 [22, 26]
*r* ^2^ fit, median [IQR]	0.97 [0.94, 0.99]	0.98 [0.96, 0.99]	0.97 [0.93, 0.99]	0.98 [0.96, 0.99]
Offsets, median [IQR]	0.41 [0.10, 0.72]	0.68 [0.48, 0.88]	0.47 [0.14, 1.20]	0.79 [0.40, 1.37]
Exponents, median [IQR]	1.00 [0.77, 0.18]	1.25 [1.11, 1.37]	0.97 [0.74, 1.20]	1.23 [0.99, 1.50]
No. of peaks detected, median [IQR]	1 [1, 2]	2 [2, 3]	1 [1, 2]	1 [1, 2]

### 2.2 Data sources

EEG data were obtained from previously collected data sets. Data from neurologically intact control individuals was from four different sources. Three were collected at the University of Utah with the involvement of two authors (K.R.L. and M.J.E.; Euler et al., 2017, 2016; Pathania et al., 2022) and one was from a publicly available dataset from the Max Planck Institute (Babayan et al., 2019). Resting EEG data for people with stroke were taken from a previous study involving one of the authors (S.C.; Cassidy et al., 2020). In summary as described by Cassidy et al., inclusion criteria for the stroke group included: radiologically confirmed stroke and age greater than 18. Exclusion criteria were as follows: contraindication to magnetic resonance imaging (MRI), substantial communication deficits, and history of cranial surgery that would introduce significant breach rhythm. For functional assessment of the stroke group, included patients were those with unilateral stroke, without contracture, significant subluxation or pain, severe neglect or apraxia, impaired level of consciousness, or a significant language deficit as previously described (Cassidy et al., 2020).

### 2.3 EEG recordings

The specific details of each dataset's recording parameters have been previously published as referenced above. Recording parameters for each dataset are summarized in Table 1. EEGs from all datasets were recorded in the resting state. Subjects were instructed to stare straight ahead with eyes open and while holding their body still. Original EEGs were recorded for 2–16 min depending on the study. Longer EEGs were manually truncated to 5 min prior to processing. Shorter EEGs (2 min) included the entire recording. Scalp EEGs were collected via 32, 62, 64 or 256 channel EEGs (electrodes and amplifiers outlined in Table 1) and were sampled at 1000 Hz (Cassidy et al., 2020; Pathania et al., 2022), 1024 Hz (Euler et al., 2016) or 2500 Hz (Babayan et al., 2019).

### 2.4 EEG processing

Raw EEG data were imported into MATLAB (MathWorks, Natick, MA, USA) and preprocessed as below using custom written MATLAB code in conjunction with EEGlab (Swartz Center for Computational Neuroscience, UCSD, San Diego, CA, USA) (development version, accessed June of 2024) (Delorme & Makeig, 2004). The data were down‐sampled to 250 Hz and high pass filtered at 0.1 Hz. Electrode data were re‐referenced to the average reference using the ‘pop‐reref’ function from EEGLab. The PREP pipeline (v0.55.5) (Bigdely‐Shamlo et al., 2015) was applied to reject excessively noisy or bad channels. The 50–60 Hz line noise was removed with the Zapline‐plus toolbox (v1.2.1) (Klug & Kloosterman, 2022) within EEG lab. Data were then decomposed into independent components using the ‘runica’ function to classify signals as cardiac rhythms, muscle artifacts, ocular movement or blinks, and then subsequently removed from the EEG signal using blind source separation via the AAR plugin for EEGlab (https://github.com/germangh/eeglab_plugin_aar/blob/master/README.md) (v131130) (Gomez‐Herrero et al., 2006) As a final pre‐processing step, any flatline channels, low‐frequency drifts, noisy channels, short‐time bursts, and incompletely repaired segments from the data were removed with the clean_rawdata plugin for EEGlab (https://github.com/sccn/clean_rawdata) (v2.8) (described in Chang et al., 2020). After undergoing artifact rejection, processed EEG data from all datasets were then subjected to fast Fourier transform using a Hamming window with 50% overlap between segments. The power spectral density was estimated using Welch's method with the number of frequency bins varying according to the sampling rate. This was performed at each channel for each participant. All code is available at github: https://github.com/margareteisfelder/Automated‐EEG‐Cleaning‐Pipeline/blob/main/README.md.

### 2.5 Spectral parameterization

Following initial processing, spectral analysis was performed using the *specparam* toolbox (Donoghue et al., 2020) running in Python (version 3). At each electrode, spectra were decomposed into periodic and aperiodic components (2–25 Hz). Settings for the specparam algorithm were as follows: peak width limits of 0.5–12, 6 maximum peaks, a minimum peak height of 0.1, and a peak threshold of 2. The quality of the of the parameterization was assessed via *r* ^2^ values provided by the algorithm and summarized for each data set in Table 1.

**FIGURE 1:**
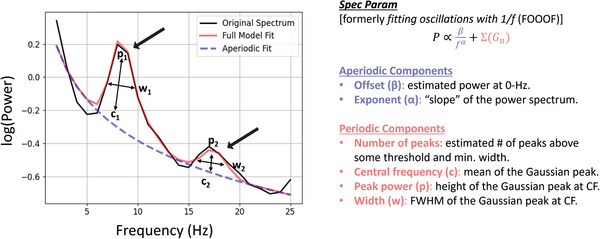
Illustration of the spectral parameterization (spec param) algorithm fit to data from a sample electrode from a control subject showing aperiodic fit (blue dashed line) and periodic activity (red peaks). At every electrode for every subject, we extract two aperiodic components: the exponent (the slope of the aperiodic fit) and the offset (the vertical position of the aperiodic fit). We also extract a number of Gaussian peaks (Gn) representing periodic activity. For every Gaussian, we obtain the central frequency, the peak power at that central frequency, and the full‐width half‐maximum (FWHM) of the Gaussian at the central frequency.

As shown in Figure 1, at each electrode we extracted two *aperiodic*, broadband components – the offset and the exponent – and several *periodic*, narrowband components for every Gaussian peak that was identified: the central frequency of the Gaussian, the peak power at the central frequency, and the full width half maximum of the Gaussian at that central frequency.

We also faced a challenge harmonizing the EEG data across these different samples owing to the different number of channels and montages used in each original data collection (see Table 1). However, we did have 28 channels that were common to all for the control datasets that could be harmonized. Note that for the high density EEG from the stroke dataset, we had to use the HydroCel geodesic sensor nets approximate 10 – 10 equivalents (see: https://www.egi.com/images/HydroCelGSN_10‐10.pdf), but to the best of our ability, these 24 channels come from the same location on the scalp in all samples and were included in analyses: F3, Fz, F4, F7, F8, FC1, FC2, FC5, FC6, C3, Cz, C4, CP1, CP2, CP5, CP6, P3, Pz, P4, P7, P8, O1, Oz, O2. Four other channels (Fp1, Fp2, T7 and T8) were harmonizable but excluded from analyses due to a lack of replicates in those regions (see Figure 4 for illustration of harmonized locations). Given that these electrode positions are similar but not identical, we also treated electrode as a random effect in statistical analyses (detailed below).

The *r* ^2^ value (representing how well the model fits the EEG power spectral data) across all channels was generally quite high: in healthy controls, median = 0.97 [IQR: 0.94, 0.99]; in adults with stroke, median = 0.97 [IQR: 0.96, 0.99]. Further, the *r* ^2^ value was generally quite high regardless of scalp location, suggesting that our processing pipeline was suitable for capturing variation in the EEG power spectrum across regions and populations: frontal median = 0.98 [IQR: 0.95, 0.99]; central median = 0.94 [IQR: 0.94, 0.99]; parietal median = 0.97 [IQR: 0.94, 0.99]; and occipital median = 0.98 [IQR: 0.95, 0.99]. We created grand average spectra for both the control groups and the stroke groups (Figure 3). Shaded ribbons represent standard error (difficult to visualize given low value in Figure 3a). Similar to our previous work, these figures combined with our *r* ^2^ values suggest that appropriate parameters were chosen, adequately capturing both periodic and aperiodic variation across the scalp and between participants.

### 2.6 Magnetic resonance imaging

**FIGURE 2:**
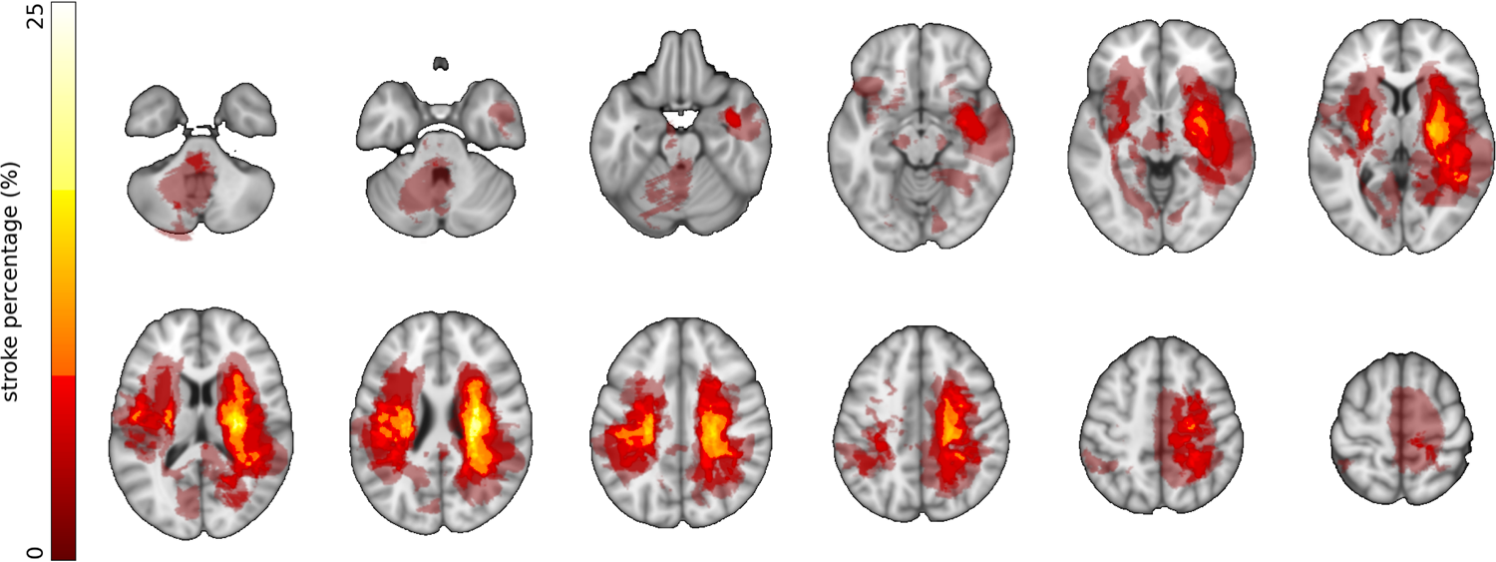
Infarct masks from 61 patients overlaid on T1‐weighted images. Brighter colours correspond to increased frequency of damage in a voxel. Right side of image corresponds to anatomical right so as to align with EEG presentations.

MRI data were available from the stroke data set and have been previously described (Cassidy et al., 2020) In brief, high resolution T1‐weighted images were acquired with a Philips Achieva 3T MRI (Philips Healthcare, Amsterdam, the Netherlands) scanner using 3D magnetization‐prepared rapid gradient echo (MPRAGE) Parameters were as follows: repetition time = 8.5 ms, echo time = 3.9 ms, slices = 150. Voxel size was 1 × 1 × 1 mm^3^. Infarct volumes were previously outlined by hand on all T1‐weighted MRI images. Individual subject MPRAGE images were linearly aligned to a reference atlas (MNI152) using *flirt* in FMRIB Software Library (FSL). The transform matrix for the subject‐to‐atlas alignment was then used to transform the stroke mask into atlas space. Stroke masks in atlas space were combined across subjects to produce a stroke frequency map representing the probability that each voxel was within the stroke mask for any individual subject (Figure 2).

### 2.7 Functional assessment

Previously obtained functional assessment scores (Box and Block Test) were utilized for our analysis. Upper extremity motor deficits were assessed via one‐time scoring on the Box and Block test measured on the same day as the EEG recording, during which subjects were asked to transfer as many blocks as possible from one side of a box to another in 60 s using the affected arm. Higher scores (number of blocks transferred) correspond to better function in the paretic limb, as previously described by Cramer et al. ( Cramer et al., 2019; Edwards et al., 2023; Milot et al., 2014)

### 2.8 Statistical analysis

**FIGURE 3:**
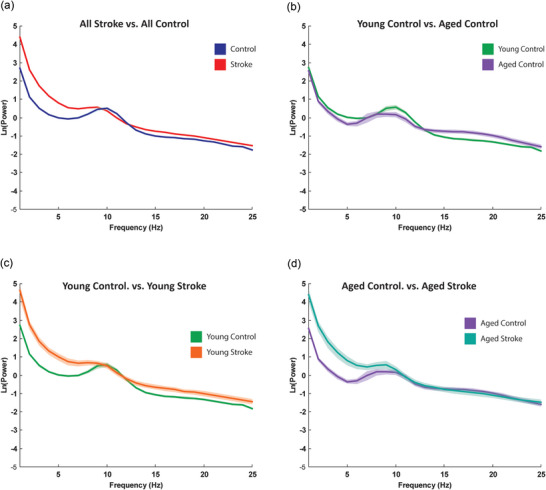
Power spectral density as generated by the specparam algorithm averaged across the scalp and across groups. Shaded areas represent standard error of the mean (SEM). Areas with minimal shading are due to small SEM. (a) Comparison of all control (*n* = 301) to all stroke individuals (*n* = 71). (b) Comparison of Aged (*n *= 56) vs. Young groups (*n *= 245) in non‐stroke individuals. (c) Comparison of Young control (*n *= 245) vs. Young Stroke (*n* = 33) individuals. (d) Comparison of Aged Control (*n* = 56) vs. Aged Stroke (*n* = 28) individuals.

To assess significant differences in power spectral density (PSD) between groups (Figure 3), we conducted cluster‐based permutation testing using the FieldTrip toolbox (Maris & Oostenveld, 2007; Oostenveld et al., 2011). Individual PSDs were averaged across channels and organized by group. PSD differences between groups (aged vs. young; stroke vs. control) were statistically tested over the frequency range of interest (1–25 Hz) using independent‐sample *t*‐statistics with a cluster‐level significance threshold of *P* < 0.05 (Monte Carlo permutations, 1000 permutations). Significant clusters identified PSD differences that survived correction for multiple comparisons. These analyses reflect a more traditional approach to analysing spectral power, combining periodic and aperiodic component into a single measure.

Subsequently, our statistical analyses separate aperiodic from periodic components and can be delineated into two main sections: comparison of healthy controls to adults with stroke, and associations of the EEG components within the stroke subgroup. In each analysis, we ran a series of mixed‐effect regression models (Bates et al., 2015; R Core Team, 2023) to address hypotheses about the aperiodic exponent of the EEG power spectrum, the number of Gaussian peaks detected, the central frequency of those peaks, and the power at those frequencies. (Note that for aperiodic components, we only analysed the exponent given our hypotheses and strong correlations we observed between offsets and exponents.)

For comparisons between controls and people with stroke, we treated region (defined as F, C, P or O electrodes) and age (as a continuous linear variable) as fixed effects. As each subject has multiple observations (i.e., one at each electrode) and electrodes were not in identical locations within each region (e.g., 10–10 vs. high density equivalents) we treated subject and electrode as random effects. This approach allows us to account for multiple sources of variation that contribute to the periodic and aperiodic signals, reducing statistical dependence in the errors of the model (Frossard & Renaud, 2020; Lohse et al., 2023). This random‐effects structure was also validated empirically, as a random effect of subject alone led to an Akaike information criterion (AIC) of −4747; adding a random effect of electrode reduced the AIC to −5900. We also explored treating region as a random effect, AIC = −5903, but found that the exponent varied systematically across regions and more variance was explained by region as a fixed effect, AIC = −5908. We did find some evidence for a non‐linear effect of age (e.g., a spline model fit the data better than a linear model), but this appeared to largely be driven by the small number of ‘middle aged’ adults in the control data. To avoid overfitting, we focused on only the simpler linear effect of age that captures most of the variance.

In the stroke subsample, models include a fixed effect of electrode hemisphere (contralesional or ipsilesional) as all participants had unilateral stroke. Central electrodes (Fz, Cz, Pz and Oz or their high density equivalents) were excluded from these analyses so that all electrodes could be defined as either ispilesional or contralesional. All models also controlled for age, sex, lesion volume and time from stroke to the EEG data collection. In all models, continuous fixed‐effects were mean centred and categorical fixed‐effects were contrast coded using orthogonal polynomials. To make the units more interpretable, age was also divided by 10 so that slopes (β_age_) show the predicted change in the outcome for 10‐year change in age.

Approximate normality of the residuals and random effects was confirmed with visual inspection of quantile–quantile normal plots. Note that *P*‐values for fixed‐effects are generally robust to violations of normality, but estimates of random effect variances are more sensitive (Schielzeth et al., 2020). Outcomes for the number of peaks and the power for each peak were square root and log‐transformed, respectively, prior to analysis to achieve approximate normality of residuals. Coefficients and 95% confidence intervals from these analyses were then back‐transformed into their original units. *P*‐values were calculated using the Satterthwaite approximation for the degrees of freedom (Kuznetsova et al., 2017) and the statistical significance for all analyses was defined as α = 0.05. Given the early phase nature of this work, we considered the cost of Type II errors (‘misses’) to be high relative to Type I errors (‘false alarms’) and did not apply a correction for multiple comparisons (Rothman, 2014). Following statistically significant omnibus main‐effects or interactions, *post hoc* tests used the estimated marginal means from the mixed‐effect model. All analyses are reported (see Supporting information, supplemental appendices for all models) with *P*‐values reported continuously, so that readers can make more conservative/corrected judgments if desired, and *P*‐values are complemented by estimates of effect size and uncertainty (Yaddanapudi, 2016).

We performed a data‐driven analysis to describe the multivariate spatial relationship between stroke location and periodic scalp‐measured electrical activity. Canonical correlation is the multivariate generalization of the more familiar Pearson correlation (Härdle & Simar, 2015). Canonical correlation identifies linear combinations (topographies) of one variable (stroke location) that is maximally correlated with the topography of another variable (exponent (α) at a given electrode). To reduce variability, increase interpretability, and given the lack of an a priori laterality hypothesis, we transformed the images such that all strokes were on the same side. This was performed by identifying strokes with centre of mass on the contralateral side and reflecting the lesion mask across the mid‐sagittal plane and the α values across the mid‐sagittal electrodes. The stroke mask matrix (dimensions = subjects × voxels) and α matrix (dimensions = subjects × electrodes) were subjected to canonical correlation analysis. Given the high dimensionality of the data, we used a sparse algorithm well suited for poorly conditioned data (Chapman & Wang, 2021). The first canonical correlation component (i.e., corresponding to the largest eigenvalue) comprises a spatial stroke mask and EEG weights. The original data, when projected onto these weights, result in maximal correlation. In this way, canonical correlation is a data driven tool for describing multivariate relationships within these data.

## 3 RESULTS

### 3.1 Age‐ and stroke‐related changes in the periodic and aperiodic components of the power spectrum

Prior work has demonstrated broad changes in power spectral density after stroke (Giaquinto et al., 1994). We first examined whether our data also demonstrated similar changes between the stroke and non‐stroke groups. Given other work showing significant spectral changes with ageing (Vlahou et al., 2014), as well as the unique relevance of stroke to the aged brain (Chen et al., 2010), we further examined spectral density across aged and young groups with and without stroke. We generated average power spectral densities for each patient (average of all electrodes across the scalp) and averaged across groups (young patients < 60 years, aged patients > 60 years, and stroke patients within each group). Spectral density comparisons for each group are illustrated in Figure 3. Consistent with work from other groups (Pathania et al., 2022; Voytek et al., 2015), we observed decreased low frequency power in the aged group (Figure 3b) when compared to the young group and was noted to be significant in our data set at 5 Hz (*P *= 0.039, cluster‐based permutation testing, Fieldtrip toolbox; Oostenveld et al., 2011). Also consistent with prior studies (Fanciullacci et al., 2017; Faught, 1993; Lu et al., 2001), stroke significantly increased power in the 1–7 Hz frequency range (Figure 3a) when compared to non‐stroke participants (*P* = 0.007). After broad spectral visualization we focused on individual differences within the periodic and aperiodic components of the power spectrum.

#### 3.1.1 Age and stroke‐related changes in the aperiodic component of the power spectrum: Exponent

**FIGURE 4:**
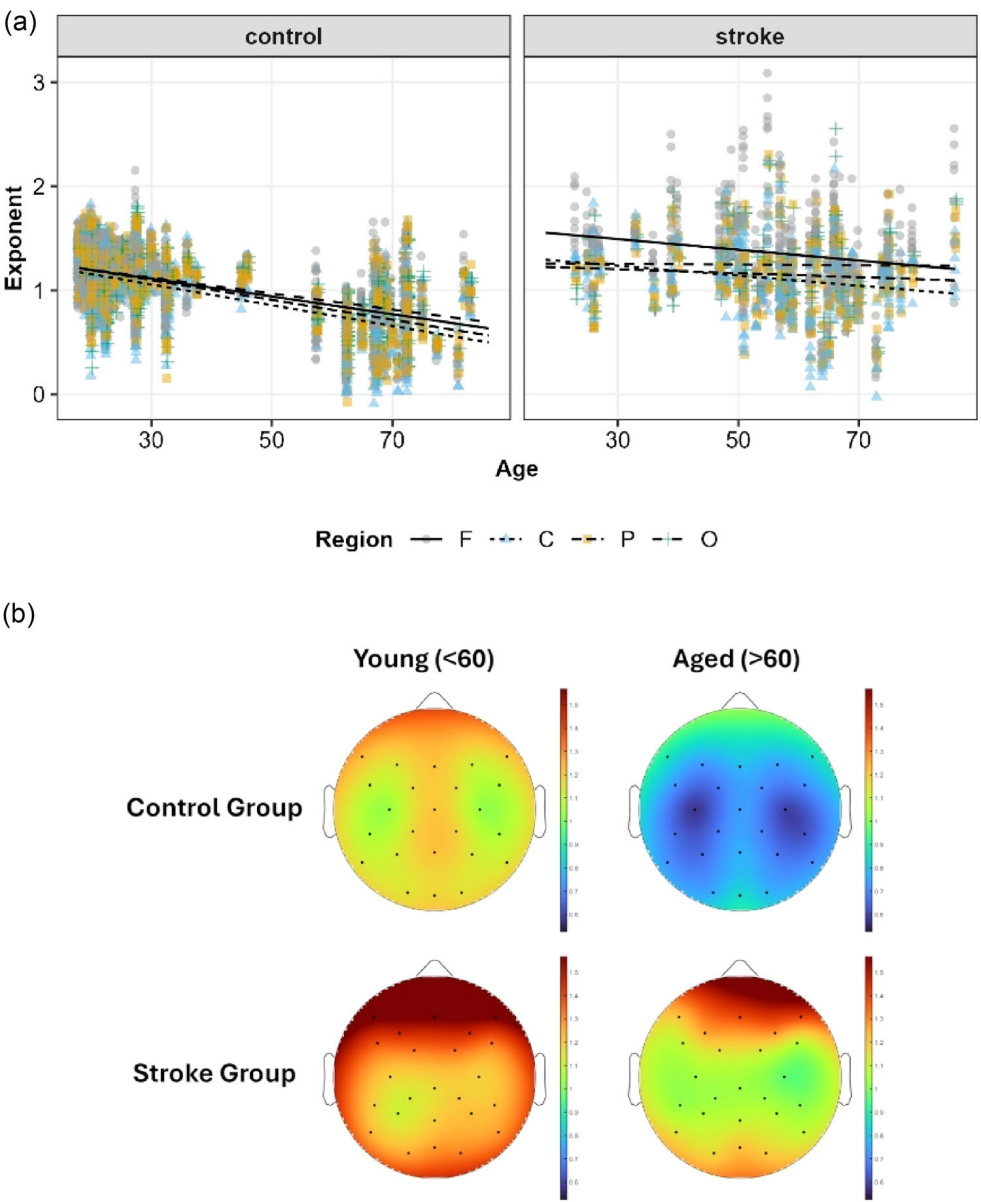
(a) Aperiodic exponents from the spectral parameterization algorithm as a function of age and scalp region (F, frontal; C, central; P, parietal; O, occipital) for neurotypical controls (left) and adults with stroke (right). Lines show estimated exponent at each age from our mixed effect model. (b) Topographies showing the distribution of average exponent across the scalp. For ease of visualization, age is dichotomized at 60 years old. Dots show electrodes included in the analysis (10–10 electrodes for controls or their nearest high‐density equivalents for adults with stroke). The colour gradient for the exponent ranges from 0.5 (dark blue) to 1.5 (dark red).

As seen in Figure 4a, there were main‐effects of Age (*F*(1, 296) = 25.8, *P *< 0.001), Group (stroke vs. neurotypical) (*F*(1, 296) = 24.3, *P *< 0.001) and Region (*F*(3, 27) = 16.4, *P *< 0.001). However, all of these effects were superseded by a Group × Region × Age interaction (*F*(3, 6762) = 14.0, *P *< 0.001). To unpack this interaction, we tested the difference between regions for the slope of age in each group. In control participants, the slope for the frontal region was β_age_ = −0.09 [95% CI: −0.10, −0.07], the central β_age_ = −0.10 [95% CI: −0.12, −0.08], the parietal β_age_ = −0.10 [95% CI: −0.11, −0.08], and the occipital β_age_ = −0.08 [95% CI: −0.09, −0.06]. In participants with stroke, these slopes were notably flatter, though only the parietal and occipital regions were significantly different from controls (*P*‐values < 0.05); the frontal β_age_ = −0.05 [95% CI: −0.10, −0.01], the central region was β_age_ = −0.05 [95% CI: −0.09, −0.01], the parietal region was β_age_ = −0.01 [95% CI: −0.06, 0.03], and the occipital region was β_age_ = −0.01 [95% CI: −0.05, −0.04]. For the full mixed effect model, see Supporting information, Supplemental Table i.

#### 3.1.2 Age‐ and stroke‐related changes in the periodic component of the power spectrum

##### Number of peaks

A mixed‐effect model examining the number of peaks identified by the spectral parameterization algorithm (Supporting information, Supplemental Table ii) showed main‐effects of Age (*F*(1, 301) = 5.6, *P *= 0.019) and Group (*F*(1, 301) = 22.8, *P *< 0.001) and a Group × Age interaction (*F*(1, 301) = 6.8, *P *= 0.009). Breaking down this interaction, there were more peaks identified in the healthy controls (mean = 2.0 [95% CI: 1.9, 2.1]) than in people with stroke (1.2 [95% CI: 0.9, 1.5]), on average. Additionally, the number of identified peaks declined with age, but this decline was more pronounced in healthy controls (β_age_ = −0.19 [95% CI: −0.25, −0.14]), and relatively flat in people with stroke (β_age_ = 0.01 [95% CI: −0.13, 0.16]).

##### Central frequency

**FIGURE 5:**
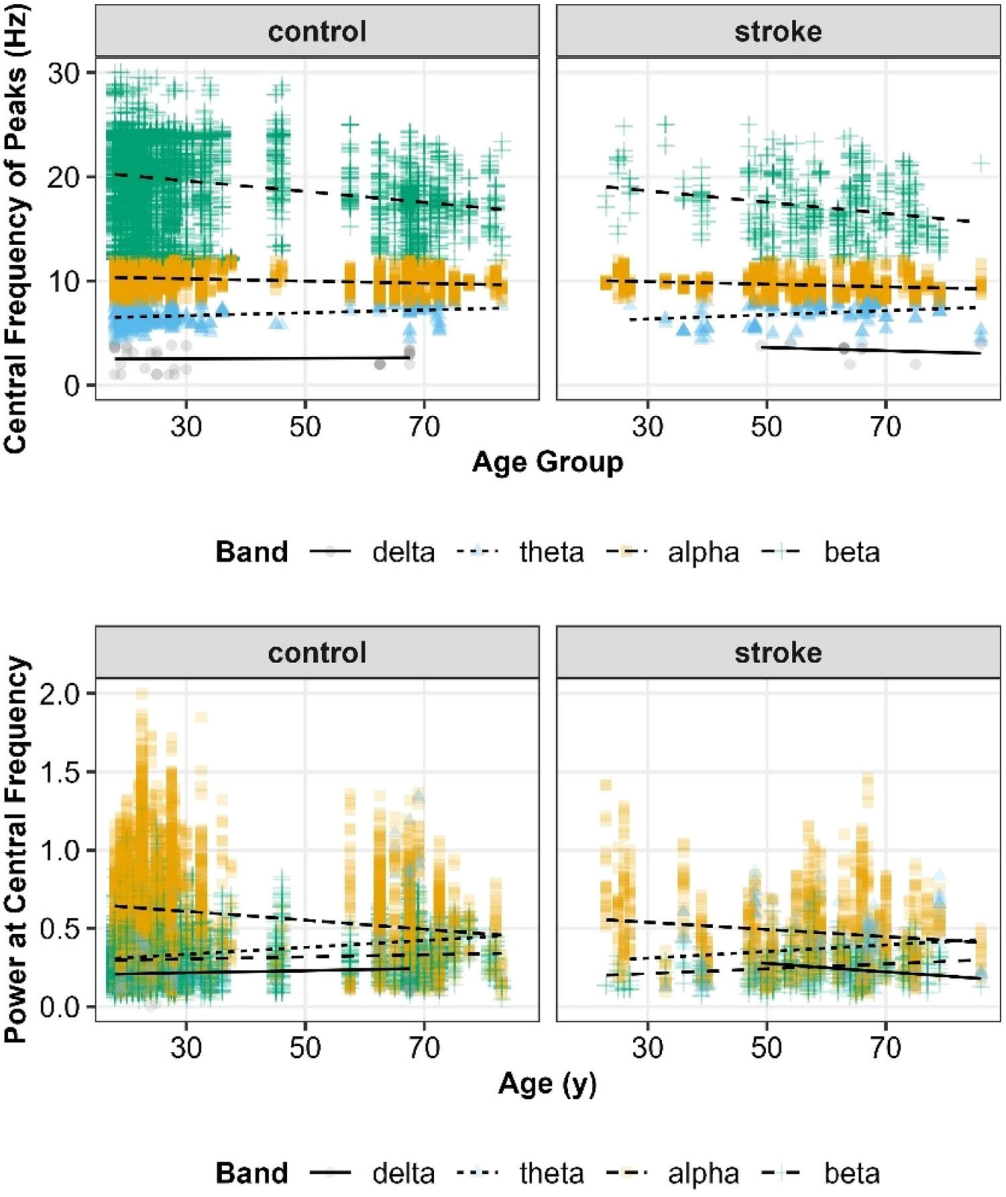
Central frequency of identified peaks (a) and the power at the central frequency (b) as a function of age and the canonical frequency bands of the EEG power spectrum.

When looking at the central frequency of the identified peaks, as shown in Figure 5a, there was a (tautological) effect of Band (*F*(2, 13420) > 100, *P *< 0.001), but also main effect of Age (*F*(1, 602) = 6.7, *P *= 0.009), an Age × Band interaction (*F*(2, 13389) = 32.9, *P *< 0.001) and a Region × Band interaction (*F*(6, 13281) = 4.9, *P *< 0.001). There were, however, no statistically significant differences between Groups (*F*(1, 662) = 1.9, *P *= 0.172), nor significant higher order interactions with Group (see Supporting information, Supplemental Table iii). As shown in Figure 5a, central frequency reliably declined with age in the beta band (β_age_ = −0.54 [95% CI: −0.67, −0.4]), but not in the alpha (β_age_ = −0.09 [95% CI: −0.20, 0.01]) or theta (β_age_ = 0.16 [95% CI: −0.05, 0.38]) band. The delta band was excluded from analysis due to limited identified peaks in that range.

##### Power at central frequency

Power at the central frequency (shown in Figure 5b) varied as a function of Band (*F*(2, 13250) > 100, *P *< 0.001), and critically showed interactions between Band × Age (*F*(2, 13263) = 33.7, *P *< 0.001) and Band × Age × Group (*F*(2, 13262) = 3.7, *P *= 0.026). The full mixed‐effects model is shown in Supporting information, Supplemental Table iv. Power tended to decline with age in the alpha band (β_age_ = −0.03 [95% CI: −0.04, −0.01]), but did not show evidence of decline in the beta (β_age_ < 0.01 [95% CI: −0.02, 0.02]) or theta (β_age_ < 0.01 [95% CI: −0.01, 0.02]) band. Control participants also generally showed more power in each frequency band compared to adults with stroke, but this difference was largest in the beta band (mean = 0.060 [95% CI: 0.004, 0.118]) and alpha band (mean = 0.047 [95% CI: −0.005, 0.102]) and much smaller in the theta band (mean = 0.014 [95% CI: −0.046, 0.078]).

### 3.2 Spatial relationships within the stroke subgroup

#### 3.2.1 Influence of lesion location and size on the aperiodic component of the power spectrum

##### Influence of lesion hemisphere on aperiodic exponent within broad cortical regions

Controlling for participants’ sex, age and days from stroke to EEG recording, there were significant main effects of Hemisphere (contralesional or ipsilesional) (*F*(1, 77) = 5.7, *P *= 0.020), Channel Region (*F*(3, 16) = 9.8, *P *< 0.001) and a Hemisphere × Region interaction (*F*(3, 1061) = 5.2, *P *= 0.001) (full details in Supporting information, Supplemental Table v). In the frontal region, contralesional electrodes had a mean exponent = 1.33 [95% CI: 1.22, 1.44], and ipsilesional electrodes had a mean = 1.30 [95% CI: 1.19, 1.42] (*P*_diff_ = 0.231). In the central region, contralesional electrodes had a mean exponent = 1.06 [95% CI: 0.94, 1.17], and ipsilesional electrodes had a mean = 1.11 [95% CI: 0.99, 1.23] (*P*_diff_ = 0.029). In the parietal region, contralesional electrodes had a mean exponent = 1.09 [95% CI: 0.97, 1.22], and ipsilesional electrodes had a mean = 1.18 [95% CI: 1.05, 1.31] (*P*_diff_ = 0.002). And finally in the occipital region, contralesional electrodes had a mean exponent = 1.19 [95% CI: 1.03, 1.34], and ipsilesional electrodes had a mean = 1.24 [95% CI: 1.08, 1.40] (*P*_diff_ = 0.162). Thus, ipsilesional electrodes generally tended to have steeper slopes than contralateral electrodes, but this difference was only statistically significant in the central and parietal regions.

##### Influence of stroke location from MRI on aperiodic exponent

**FIGURE 6:**
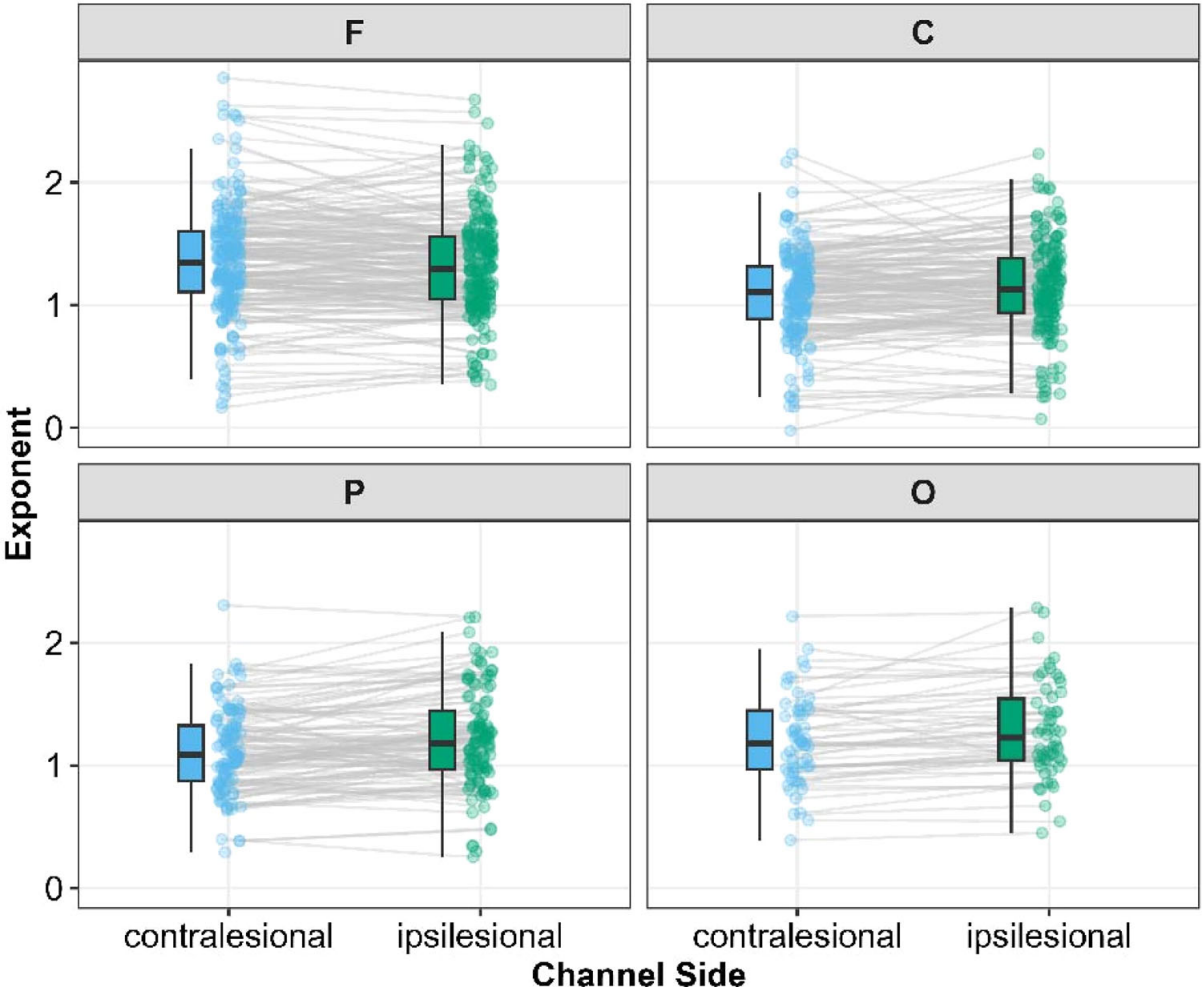
Aperiodic exponents as a function of channel region (F, frontal; C, central; P, parietal; O, occipital) and channel side (either ipsilesional or contralesional).

As noted in Figure 6, we observed a significant relationship between stroke hemisphere and aperiodic activity (exponent). This analysis is limited to hemispheric regions. We next sought to more precisely quantify the relationship between exponent and lesion location using MRI data from the participants within the stroke group.

**FIGURE 7:**
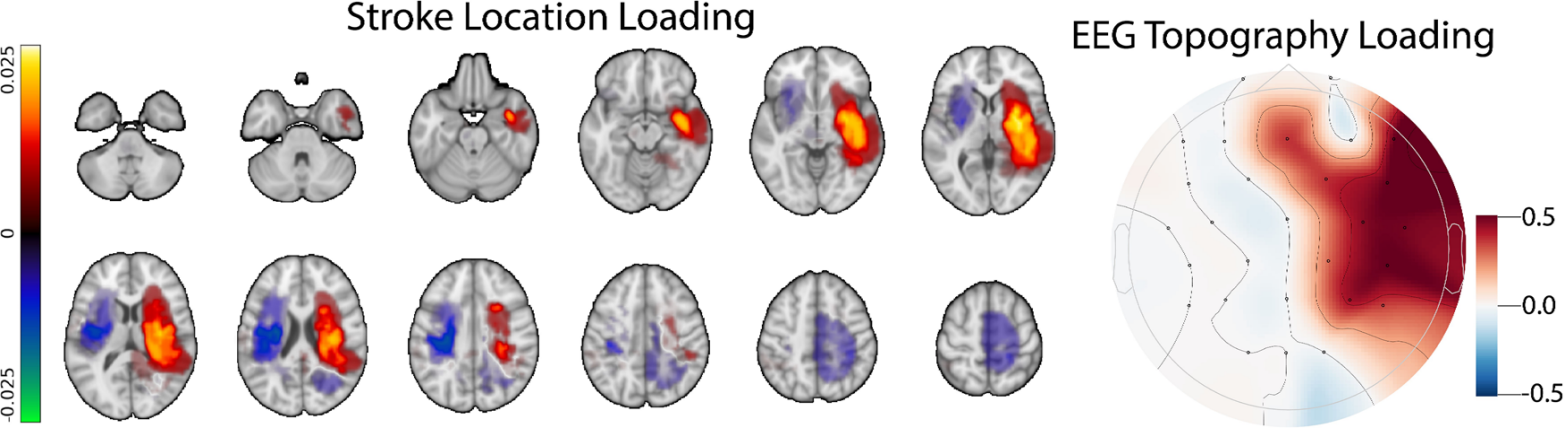
Stroke spatial topography (left) and EEG α topography (right) corresponding to the first canonical correlation between stroke location and EEG beta coefficient in all 61 stroke patients with MRI data. Strokes were aligned so that they all appear on the right; the EEG was similarly transposed across the mid‐sagittal plane to define a stroke side (right) and non‐stroke side (left). This result shows a strong correlation between deep strokes and increased slope of the EEG signal over ispilesional frontal electrodes.

We used canonical correlation to examine the multivariate relationship between stroke topography and topography of disrupted EEG as measured by the exponent (α) (Figure 7). The first canonical correlation is defined by a stroke topography that is maximally correlated with an EEG topography. Recall that, absent an a priori laterality hypothesis, we transformed images into stroke side vs. non‐stroke side instead of anatomical left vs. right. The first canonical correlation identified a stroke topography maximally loaded in the deep white matter, which also encompasses deep grey nuclei. In contrast, stroke topography was negatively loaded in the contralesional brain. The EEG topography was maximally loaded ispilesionally with maximal values in frontal and pre‐frontal electrodes. This demonstrates that the presence of a stroke in the deep structures of the brain is positively related to changes in EEG α over the ipsilateral frontal scalp with minimal impact on the contralateral EEG activity. This indicates that deep strokes manifest as steeper EEG spectra over the affected side.

#### 3.2.2 Influence of lesion location on the periodic component of the power spectrum

##### Number of peaks and central frequency

There were no statistically significant differences in the number of peaks or the central frequencies of peaks as a function of ipsilesional vs. contralesional hemisphere (see Supporting information, Supplemental Tables vi and vii). Thus, the spectral parameterization algorithm appeared to be identifying peaks on the contralesional and ipsilesional side in similar ways, and we can more robustly interpret differences in power for the observed peaks.

##### Narrowband power at central frequency

**FIGURE 8:**
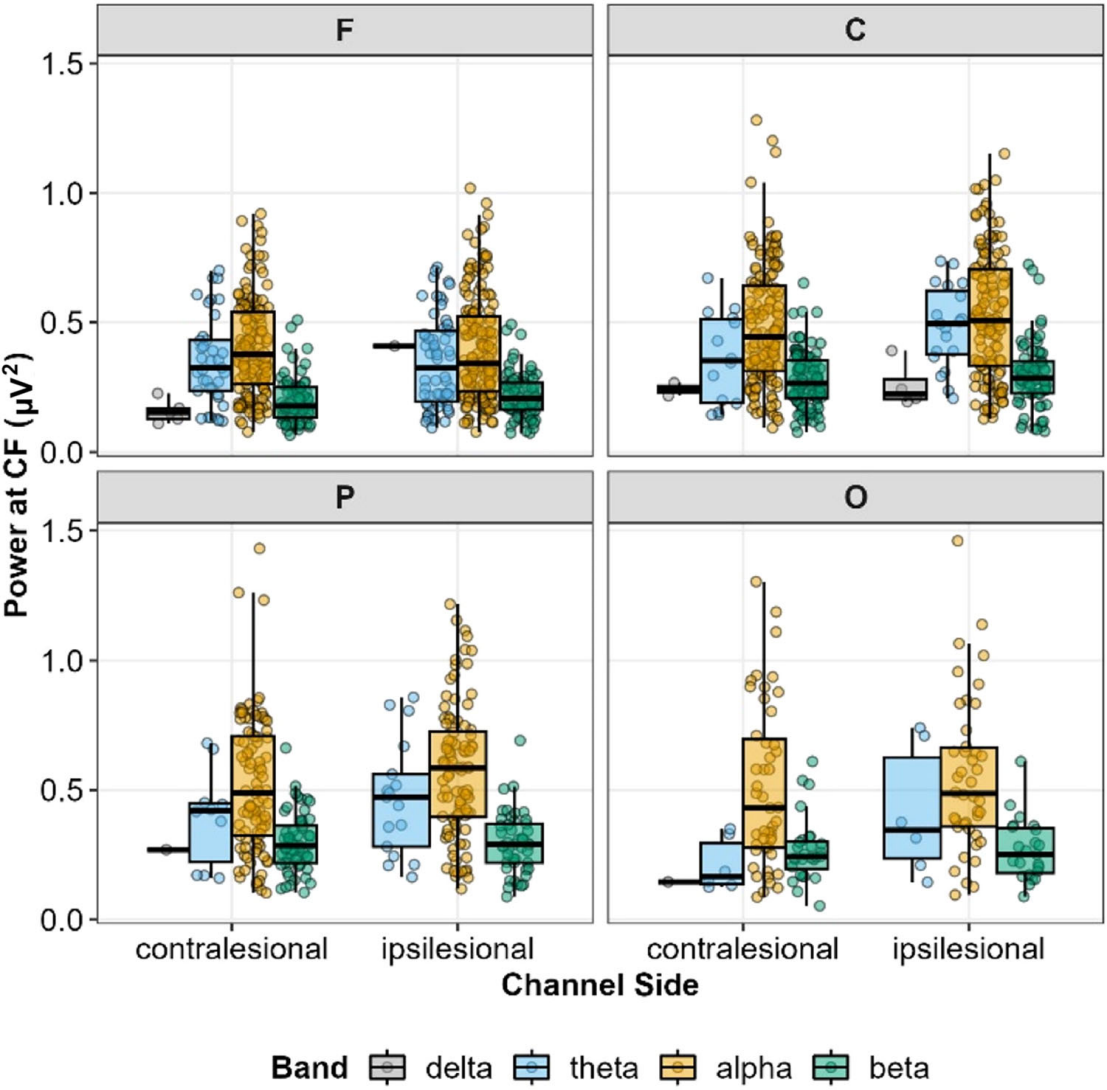
Narrowband power as a function of the canonical frequency bands (delta, grey; theta, blue; alpha, gold; beta, green), scalp region (frontal, central, parietal or occipital), and channel side relative to the lesion (contralesional or ipsilesional).

Controlling for participants sex, age, lesion volume and days from stroke to EEG recording, there were statistically significant main effects of Band (*F*(2, 1377) = 218.8, *P *< 0.001) and Region (*F*(3, 28) = 18.1, *P *< 0.001) on narrow‐band power (Figure 8). There were also statistically significant interactions of Band × Region (*F*(6, 1346) = 2.96, *P *= 0.007) and Hemisphere × Region (*F*(3, 1364) = 3.48, *P *= 0.015). No other effects were statistically significant (*P*‐values > 0.070) (see supporting information, Supplemental Table viii). *Post hoc* tests of these interactions revealed that narrowband power was generally lower at the frontal electrodes compared to central and parietal electrodes, and these effects were more pronounced on the ipsilesional side (i.e., contralesional F–C, *P *= 0.014; F–P; *P *= 0.002; F–O, *P *= 0.891; ipsilesional F–C, *P *< 0.001; F–P, *P *< 0.001; F–O = 0.017). Further, power was generally highest in the alpha band, but the strength of this effect depended on the specific region of the scalp. For instance, alpha power was reliably higher than beta power across all regions (*P*‐values < 0.001), but alpha power was not significantly different from theta power in the frontal (*P *= 0.122), central (*P *= 0.363) or parietal region (*P *= 0.068), only reaching the level of a statistically significant difference in the occipital region (*P *< 0.001). As with previous analyses, the delta band was excluded due to a limited number of peaks identified in the delta band.

### 3.3 Relationship between aperiodic activity and motor outcomes

**FIGURE 9:**
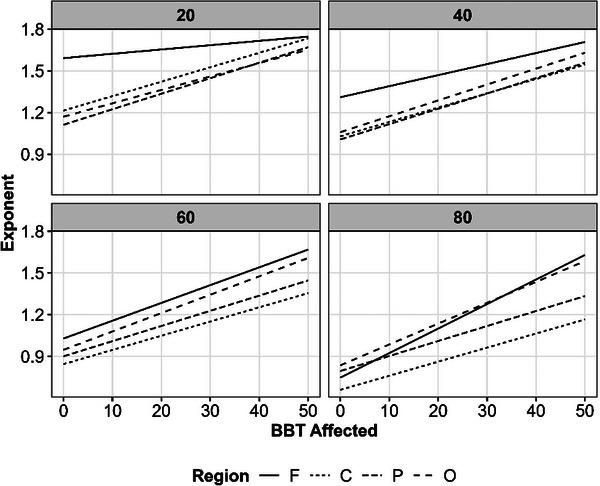
Aperiodic exponents as a function of age, region, and box and block test (BBT) score (higher values = better performance). Lines show the predicted relationship between BBT and the exponent at four example ages to illustrate the Age × Region × BBT interaction, but note that both Age and BBT were treated as continuous linear variables in the model.

We sought to examine whether abnormalities in aperiodic activity (recently shown to contribute to post‐stroke spectral slowing; Johnston et al., 2023) are associated with motor outcomes after stroke. We examined the relationship between exponent and Box and Block scores in the stroke group. We observed a relatively linear correlation between performance on Box and Block testing and exponent, with steeper slopes being associated with better performance (Figure 9).

In a model controlling for sex, days to enrolment and lesion volume, we saw a main effect of BBT for the affected side (*F*(1, 37) = 7.9, *P *= 0.008), Electrode Region (*F*(3, 19) = 12.3, *P *< 0.001) and Hemisphere (*F*(1, 52) = 6.1, *P *= 0.017). However, these effects were further complicated by two‐way interactions of Age × Region (*F*(3, 648) = 6.0, *P *< 0.001) and Region x Hemisphere (*F*(3, 654) = 3.2, *P *= 0.024), and by a further three‐way interaction of BBT × Age × Region (*F*(3, 647) = 4.8, *P *= 0.002). We present this three‐way interaction in Figure 9. Consistent with the main effect of BBT, there was generally a positive relationship between BBT and the exponent. However, the strength of this relationship varied by region and age. Due to the large number of possible *post hoc* comparisons, we did not compare all regions. However, we tried to illustrate the heterogeneity of this association by plotting the model's predictions at every region for four candidate ages (20, 40, 60 and 80 years). Broadly speaking, the association between the BBT and the exponent was stronger in older adults.

## 4 DISCUSSION

Here we examined the differences in periodic and aperiodic activity in healthy young and old individuals as well as younger and older individuals with stroke. In keeping with prior work, we observed decreased low frequency power with age and increased low frequency power spectral density after stroke (Figure 3). We next noted a significant age‐related decrease in power spectral slope (consistent with prior work; Johnston et al., 2023) (Figure 4). Interestingly, this age‐dependent decrease in spectral slope was significantly attenuated in individuals with stroke (Figure 4) resulting in aged individuals with stroke having a significantly steeper spectral slope than aged control individuals. Within the periodic component of the spectrum, we found that stroke was associated with fewer frequency‐specific peaks, and this effect was greatest in young individuals. We also noted effects of both age and stroke on central frequency and power of periodic activity (Figure 5). With regard to lesion location, we noted power spectra were steeper (i.e., larger exponents) on the ipsilesional hemisphere (Figure 6) and that deep lesions had a disproportionate impact on exponent (Figure 7). Finally we noted a complex interaction between performance on box and block test, age and scalp region. Although the precise pattern varied by region, we broadly saw a stronger association with number of blocks placed and the exponent of the power spectrum (Figure 9).

Prior work has demonstrated several EEG changes associated with stroke. Stroke is classically associated with spectral slowing characterized by diminished high frequency (beta/gamma) activity (Chu et al., 2015) as well as increased low frequency power (Fanciullacci et al., 2017; Faught, 1993; Lu et al., 2001). Delta (1–4 HZ) power in particular has been reported to correlate with both injury size and motor recovery (Cassidy et al., 2020). Recent work demonstrated that ‘spectral slowing’ observed after chronic stroke is not merely a reflection of changes in periodic activity, but rather a combination of periodic shifts and changes in aperiodic activity (spectral slope) (Johnston et al., 2023). This work suggested that increased low frequency power observed after stroke was at least partially explained by a shift in aperiodic activity toward lower frequencies as quantified by greater aperiodic exponent. This work built on prior animal work suggesting that increased low power activity was a reflection of a change in aperiodic activity and correlated to outcomes in a rat model of stroke (Leemburg et al., 2018). Our data support these findings with a similar observation that stroke is associated with significant changes in aperiodic activity as reflected by a greater aperiodic exponent in individuals with stroke. Here we note a significant age‐dependent effect on aperiodic activity with young individuals after stroke having more similar exponents to those without stroke while older adults with stroke have much higher exponents than those without stroke. This change in exponent is especially striking as it is the opposite of the typical direction of exponent change with ageing. Together, these findings raise the intriguing possibility that stroke‐associated changes in aperiodic activity may reflect a unique pathology of ageing. It is well‐established that older individuals with stroke have worse outcomes (Strbian et al., 2013) and diminished functional recovery (Saposnik et al., 2008). It is interesting to speculate that age‐specific disruptions in neuronal activity may contribute to diminished plasticity and recovery in aged individuals after stroke. This is particularly intriguing given our finding that higher slopes correlate with better functional performance in older individuals but not younger individuals.

As others have noted, the physiological underpinnings of aperiodic activity and aperiodic slope in spectral parameterization of EEG recordings are still being elucidated, but a significant body of evidence has suggested that these slope changes may reflect changes in the balance of network level excitatory and inhibitory activity. Computational work has shown that flatter slope is associated with an increased ratio of excitation to inhibition (Gao et al., 2017; Lombardi et al., 2017; Trakoshis et al., 2020). Additional experimental work has demonstrated that the slope of hippocampal field potentials correlates to the relative density of excitatory vs. inhibitory synapses near the recording electrode (Gao et al., 2017). Additionally, global inhibition with propofol (a GABAergic anaesthetic agent) is correlated with steeping of spectral slope as measured by electrocorticography (Gao et al., 2017).

Stroke is associated with well‐characterized changes in the ratio between excitation and inhibition. During the hyper‐acute phase of stroke, excitotoxicity is hypothesized to directly contribute to ischaemic cell death (reviewed by Neves et al., 2023). In the subacute phase after stroke, animal studies have demonstrated increased tonic inhibition which likely limits recovery (Clarkson et al., 2010) while potentially reducing excitotoxicity. Work in both animals (Carmichael, 2012; Schiene et al., 1996) and humans (Blicher et al., 2015) has further suggested that the longer post‐stroke phase is associated with a shift toward more excitatory network behaviour and that this shift is important for promoting plasticity. If indeed spectral slope reflects alterations in excitation–inhibition balance, our data would suggest that in older individuals, the chronic phase of stroke is associated with a shift toward greater inhibition (steeper aperiodic slope). It is interesting to speculate that the degree of this shift may reflect varying levels of recovery, with those having the greatest shift demonstrating the best outcomes. It is important to note, however, as others have speculated (Johnston et al., 2023), that changes in slope seen after stroke may be driven by other mechanisms as well, including disruption of structural connectivity between cerebral regions. Ageing itself is also associated with changes in excitation/inhibition ratio. Diminished inhibitory activity has been noted with normal ageing in both rodents (Schmidt et al., 2010) and humans (Bhandari et al., 2016). This shift may be reflected by the gradual flattening of the exponent observed in our study and others’ (Voytek et al., 2015). Chronic stroke may negate this age‐related increase in excitation/inhibition ratio as reflected by steeper spectral slope, though this clearly does not occur in an adaptive manner.

It is interesting to note that the ‘steepening’ or increase in exponent we observed in aged individuals with stroke causes the observed slopes to resemble more closely those of the younger population (before they have undergone an age‐related decline in exponent). This is not to suggest, however, that stroke is in some way associated with improved cognitive performance. Indeed, a wide body of literature has shown that stroke is associated with accelerated cognitive decline (El Husseini et al., 2023) as well as changes in mood (Liu et al., 2023). Additionally, stroke itself is an independent risk factor for dementia (Koton et al., 2022; Pendlebury et al., 2019). We speculate that disruption in aperiodic activity in either direction – flattening with slope with age or steepening of slope in aged stroke patients – may be associated with cerebral dysfunction and that it reflects a more general disruption in neuronal activity. It is especially interesting to consider that the aged brain may be uniquely vulnerable to this kind of disruption after stroke.

Another interesting observation from our work is the relationship between lesion location and exponent. We observed the greatest effect on the exponent in ipsilesional electrodes (electrodes nearest the stroke) (Figure 6). While it is unsurprising that we observed the greatest effect of stroke nearest the stroke, it is compelling to consider the more widespread nature of the effect. Contralateral electrodes from the stroke group still exhibited abnormalities in the exponent, suggesting stroke mediated disruption of aperiodic network activity throughout the brain. Prior work employing both human functional imaging (Olafson et al., 2021; Siegel et al., 2016) and animal studies (Bauer et al., 2014; Bice et al., 2022) has shown that focal stroke is associated with widespread disruption in functional network connectivity and that these disconnections may be superior predictors of deficit and recovery within certain functional domains than lesion location. Disruption of these broad networks, particularly thalamocortical networks (Malekmohammadi et al., 2015), may be a primary driver of the widespread disruption of aperiodic activity. Our observation that deep lesions had the greatest influence on cortical aperiodic activity further suggests a role for network disruption as a driver of widespread (non‐focal) cortical activity changes after stroke (Figure 7). This is particularly intriguing to consider given the broad role thalamocortical connections driving feed‐forward inhibition of cortical circuits (Lee et al., 2020; Swadlow, 2003) while playing an integral role in consciousness (Seo et al., 2022) and memory (Bolkan et al., 2017). The robust influence of deep lesions on aperiodic slope we observe may represent disruption of cortical excitation–inhibition balance driven by damaged thalamocortical connectivity.

We also observed several changes within the periodic components of the spectrum in both stroke and non‐stroke individuals. First, we noted fewer total peaks in the stroke group than the control group. This was most prominent when comparing young patients. Fewer identified peaks may suggest changes in spectral density are driven disproportionally by changes in aperiodic activity after stroke. We also observed a general slowing of central frequency within peaks with ageing particularly in higher frequency bands. Central frequency in the alpha band was slower in individuals with stroke (Figure 5). This influence of stroke on alpha central frequency has previously been observed (Johnston et al., 2023). It is intriguing that, as with aperiodic activity, we note some baseline influence of age; however, it is not as robust as the influence of stroke and age on aperiodic activity – again recapitulating prior work suggesting that aperiodic changes are a significant driver of post‐stroke changes in spectral activity.

This work has several strengths including the significant number of participants, the inclusion of motor outcomes and the wide range of ages. The study also has several important limitations. First, stroke is intrinsically a heterogenous disease. Our data reflect a variety of stroke locations and severities. Future work is needed to better characterize how differences in stroke location influence EEG activity. Our data were also obtained across a wide range of time points post‐stroke. Although we controlled for this variable in our analyses, it still limits our ability to determine what is necessarily a reflection of chronic stroke vs. ongoing acute changes in stroke. As an initial step, at least, our analysis did not find a relationship between time post‐stroke and exponent. Our work also only examined the relationship between exponent and one test of manual dexterity (box and block). Future work should focus on a wider array of post‐stroke functional testing. Another limitation was the relatively higher number of young and older strokes with fewer strokes in the ‘middle age contingent’. Future work will focus on adding additional ages to our analysis. Additionally, the strokes in our dataset were generally smaller strokes, and information regarding other relevant factors such as concurrent neuro‐active medications was limited. It will be important to explore these findings in larger strokes and compare the influence of additional factors such as the use of neuroactive medications. Finally, future work should focus on correlating these measures to objective measures of outcomes including both motor and psycho‐cognitive tests. Despite these limitations, our work builds substantially on prior work describing changes in cortical activity after stroke and characterizes a key age‐dependent effect of stroke on aperiodic cortical activity. It also provides a starting point for a more granular examination of these findings in different stroke pathologies and time points as well as in non‐vascular neurological pathologies.

In conclusion, here we show that patients with stroke exhibit significant chronic changes in both the periodic and aperiodic components of the power spectrum. Our work replicates recent work (Johnston et al., 2023) demonstrating a shift in aperiodic activity (slope) as a significant contributor to cortical spectral changes observed after stroke. We also expand on past work by leveraging multiple large data sets to specifically examine the influence of age on aperiodic activity. We found that the influence of stroke on exponent was primarily observed in older individuals, and that stroke had minimal effect on exponent in younger individuals. Additionally, we found the greatest influence on exponent was from deep strokes, and while the greatest effect was observed near the stroke, brain‐wide changes in periodic activity were observed. Finally, our work suggests that slope in aged but not young individuals may be a relevant biomarker of motor performance. We feel these findings will add significant value to future translational work focused on manipulations to brain activity after stroke including studies focused on neurostimulation or pharmacological interventions targeting excitation/inhibition. Critically, our work highlights the necessity of controlling for age when investigating cortical activity patterns after stroke.

## AUTHOR CONTRIBUTIONS

Asher J. Albertson, Jin‐Moo Lee, and Keith R. Lohse conceived the study, designed and performed analyses, generated figures, and drafted and revised the manuscript. Eric C. Landsness, Margaret Eisfelder, Matthew R. Brier, Bradley Judge, and Brittany M. Young curated and analyzed data, generated figures, and contributed to manuscript revision. Matthew J. Euler and Steven C. Cramer contributed original datasets, provided resources, assisted with analyses, and revised the manuscript. All authors approved the final manuscript. All authors have read and approved the final version of this manuscript and agree to be accountable for all aspects of the work in ensuring that questions related to the accuracy or integrity of any part of the work are appropriately investigated and resolved. All persons designated as authors qualify for authorship, and all those who qualify for authorship are listed.

## CONFLICT OF INTEREST

None declared.

## Supporting information



Supplemental Tables i–ix.

## Data Availability

Code for the EEG processing pipeline are available from: https://github.com/margareteisfelder/Automated‐EEG‐Cleaning‐Pipeline/blob/main/README.md and summary data and code for all statistical analyses are available from: https://github.com/keithlohse/stroke_EEG.
