# Supplementary material for: Stroke and motor outcomes are associated with regional and age‐specific changes in periodic and aperiodic cortical activity
Source: Exp Physiol. 2025 Sep 24;111(3):1210–27. doi: 10.1113/EP093171 (PMC12949189; doi:10.1113/EP093171)
Supplement: Supplementary file 1 — Supplemental Tables i–ix. [file EPH-111-1210-s001.docx]

**Supplemental Appendix**

**Contents**

[Supplemental Table i. Exponent of the power spectrum as an effect of linear age, scalp region and group (stroke versus control). 2](#_Toc200455948)

[Supplemental Table ii. Number of periodic peaks identified by spec_param as a function of linear age, scalp region and group (stroke versus control). 4](#_Toc200455949)

[Supplemental Table iii. Central frequency of periodic peaks identified by spec_param as a function of linear age, scalp region and group (stroke versus control). 6](#_Toc200455950)

[Supplemental Table iv. Power at the central frequency of periodic peaks identified by spec_param as a function of linear age, scalp region and group (stroke versus control). 9](#_Toc200455951)

[Supplemental Table v. Exponent from the power spectrum in people with stroke as a function of scalp region and hemisphere (ipsilesional or contralesional) controlling for age, sex, and time since stroke. 12](#_Toc200455952)

[Supplemental Table vi. Number of periodic peaks identified by spec_param in people with stroke as a function of scalp region hemisphere (ipsilesional or contralesional), and frequency band, controlling for age, sex, and time since stroke. 14](#_Toc200455953)

[Supplemental Table vii. Central frequency of periodic peaks identified by spec_param in people with stroke as a function of scalp region, hemisphere (ipsilesional or contralesional), and frequency band controlling for age, sex, and time since stroke. 17](#_Toc200455954)

[Supplemental Table viii. Power at the central frequency of periodic peaks identified by spec_param in people with stroke as a function of scalp region, hemisphere (ipsilesional or contralesional), and frequency band controlling for age, sex, and time since stroke. 20](#_Toc200455955)

[Supplemental Table ix. Associations between the Box and Block Test (BBT) and the exponent of the power spectrum in people with stroke as a function of scalp region, hemisphere (ipsilesional or contralesional), and frequency band controlling for age, sex, and time since stroke. 23](#_Toc200455956)

# Supplemental Table i. Exponent of the power spectrum as an effect of linear age, scalp region and group (stroke versus control).

Linear mixed model fit by maximum likelihood . t-tests use Satterthwaite's method [

lmerModLmerTest]

Formula: Exponent ~ age.c * region * group + (1 | subID) + (1 | Channels)

Data: COMB

Control: lmerControl(optimizer = "bobyqa", optCtrl = list(maxfun = 5e+05))

AIC BIC logLik deviance df.resid

-6555.9 -6425.4 3296.9 -6593.9 7061

Scaled residuals:

Min 1Q Median 3Q Max

-6.8196 -0.5485 -0.0048 0.5392 7.7140

**Random effects:**

Groups Name Variance Std.Dev.

subID (Intercept) 0.060174 0.2453

Channels (Intercept) 0.002237 0.0473

Residual 0.019029 0.1379

Number of obs: 7080, groups: subID, 295; Channels, 24

**Fixed effects:**

Estimate Std. Error df t value Pr(>|t|)

(Intercept) 1.139e+00 2.807e-02 2.956e+02 40.579 < 2e-16 ***

age.c -5.980e-02 1.178e-02 2.963e+02 -5.075 6.86e-07 ***

region1 9.666e-02 1.602e-02 2.663e+01 6.035 2.04e-06 ***

region2 -6.408e-02 1.719e-02 2.663e+01 -3.728 0.000919 ***

region3 -4.333e-02 1.911e-02 2.663e+01 -2.267 0.031716 *

group1 -1.284e-01 2.604e-02 2.963e+02 -4.930 1.37e-06 ***

age.c:region1 -8.451e-03 2.128e-03 6.762e+03 -3.971 7.24e-05 ***

age.c:region2 -1.395e-02 2.284e-03 6.762e+03 -6.106 1.08e-09 ***

age.c:region3 2.472e-03 2.540e-03 6.762e+03 0.973 0.330458

age.c:group1 -2.933e-02 1.178e-02 2.963e+02 -2.489 0.013368 *

region1:group1 -7.873e-02 4.703e-03 6.762e+03 -16.741 < 2e-16 ***

region2:group1 1.295e-02 5.046e-03 6.762e+03 2.566 0.010311 *

region3:group1 3.928e-02 5.612e-03 6.762e+03 6.999 2.83e-12 ***

age.c:region1:group1 1.244e-02 2.128e-03 6.762e+03 5.846 5.26e-09 ***

age.c:region2:group1 3.551e-03 2.284e-03 6.762e+03 1.555 0.120041

age.c:region3:group1 -8.766e-03 2.540e-03 6.762e+03 -3.451 0.000561 ***

---

Signif. codes: 0 ‘***’ 0.001 ‘**’ 0.01 ‘*’ 0.05 ‘.’ 0.1 ‘ ’ 1

**Type III Analysis of Variance Table with Satterthwaite's method**

Sum Sq Mean Sq NumDF DenDF F value Pr(>F)

age.c 0.4901 0.49006 1 296.3 25.7541 6.859e-07 ***

region 0.9343 0.31145 3 26.6 16.3674 3.138e-06 ***

group 0.4625 0.46254 1 296.3 24.3077 1.370e-06 ***

age.c:region 1.2305 0.41017 3 6762.0 21.5556 6.860e-14 ***

age.c:group 0.1179 0.11786 1 296.3 6.1939 0.01337 *

region:group 5.5800 1.86000 3 6762.0 97.7479 < 2.2e-16 ***

age.c:region:group 0.7969 0.26562 3 6762.0 13.9591 4.531e-09 ***

---

Signif. codes: 0 ‘***’ 0.001 ‘**’ 0.01 ‘*’ 0.05 ‘.’ 0.1 ‘ ’ 1

# Supplemental Table ii. Number of periodic peaks identified by spec_param as a function of linear age, scalp region and group (stroke versus control).

Linear mixed model fit by maximum likelihood . t-tests use Satterthwaite's method [lmerModLmerTest]

Formula: sqrt(peak_count + 1) ~ age.c * group * region + (1 | subID) + (1 | Channels)

Data: PEAK_COUNT

AIC BIC logLik -2*log(L) df.resid

2286.5 2417.0 -1124.3 2248.5 7061

Scaled residuals:

Min 1Q Median 3Q Max

-4.6393 -0.4603 0.0225 0.6193 3.6278

**Random Effects:**

Groups Name Variance Std.Dev.

subID (Intercept) 0.048548 0.22034

Channels (Intercept) 0.001904 0.04364

Residual 0.070895 0.26626

Number of obs: 7080, groups: subID, 295; Channels, 24

**Fixed Effects:**

Estimate Std. Error df t value Pr(>|t|)

(Intercept) 1.579e+00 2.590e-02 2.843e+02 60.966 < 2e-16 ***

age.c -2.572e-02 1.087e-02 3.011e+02 -2.366 0.01863 *

group.L -1.623e-01 3.398e-02 3.011e+02 -4.776 2.79e-06 ***

region.L -2.894e-03 2.417e-02 3.711e+01 -0.120 0.90534

region.Q -2.048e-02 2.301e-02 3.711e+01 -0.890 0.37917

region.C 1.675e-02 2.179e-02 3.711e+01 0.769 0.44691

age.c:group.L 4.011e-02 1.538e-02 3.011e+02 2.608 0.00955 **

age.c:region.L 1.001e-02 5.914e-03 6.762e+03 1.693 0.09046 .

age.c:region.Q -2.810e-03 5.631e-03 6.762e+03 -0.499 0.61781

age.c:region.C -2.827e-03 5.332e-03 6.762e+03 -0.530 0.59596

group.L:region.L -2.084e-02 1.848e-02 6.762e+03 -1.128 0.25942

group.L:region.Q -1.862e-02 1.760e-02 6.762e+03 -1.058 0.29012

group.L:region.C 4.818e-03 1.666e-02 6.762e+03 0.289 0.77249

age.c:group.L:region.L 1.843e-02 8.364e-03 6.762e+03 2.203 0.02763 *

age.c:group.L:region.Q -6.421e-03 7.963e-03 6.762e+03 -0.806 0.42007

age.c:group.L:region.C 4.950e-04 7.541e-03 6.762e+03 0.066 0.94766

---

Signif. codes: 0 ‘***’ 0.001 ‘**’ 0.01 ‘*’ 0.05 ‘.’ 0.1 ‘ ’ 1

**Type III Analysis of Variance Table with Satterthwaite's method**

Sum Sq Mean Sq NumDF DenDF F value Pr(>F)

age.c 0.39679 0.39679 1 301.1 5.5968 0.018626 *

group 1.61735 1.61735 1 301.1 22.8134 2.793e-06 ***

region 0.13051 0.04350 3 37.1 0.6136 0.610484

age.c:group 0.48234 0.48234 1 301.1 6.8036 0.009552 **

age.c:region 0.30491 0.10164 3 6761.9 1.4336 0.230852

group:region 0.14944 0.04981 3 6761.9 0.7026 0.550354

age.c:group:region 0.53577 0.17859 3 6761.9 2.5191 0.056199 .

---

Signif. codes: 0 ‘***’ 0.001 ‘**’ 0.01 ‘*’ 0.05 ‘.’ 0.1 ‘ ’ 1

# Supplemental Table iii. Central frequency of periodic peaks identified by spec_param as a function of linear age, scalp region and group (stroke versus control).

Linear mixed model fit by maximum likelihood . t-tests use Satterthwaite's method [

lmerModLmerTest]

Formula: CF ~ band * age.c * group * region + (1 | subID) + (1 | Channels)

Data: PEAK_BAND %>% filter(band != "delta")

Control: lmerControl(optimizer = "bobyqa", optCtrl = list(maxfun = 5e+05))

AIC BIC logLik deviance df.resid

62872.8 63255.7 -31385.4 62770.8 13414

Scaled residuals:

Min 1Q Median 3Q Max

-3.7981 -0.3687 0.0137 0.4760 4.6921

**Random effects:**

Groups Name Variance Std.Dev.

subID (Intercept) 0.935794 0.96736

Channels (Intercept) 0.009436 0.09714

Residual 5.922038 2.43352

Number of obs: 13465, groups: subID, 295; Channels, 24

**Fixed effects:**

Estimate Std. Error df t value Pr(>|t|)

(Intercept) 1.169e+01 1.383e-01 6.437e+02 84.508 < 2e-16 ***

band.L 8.172e+00 1.865e-01 1.344e+04 43.818 < 2e-16 ***

band.Q 2.090e+00 1.210e-01 1.343e+04 17.266 < 2e-16 ***

age.c -1.569e-01 6.047e-02 6.021e+02 -2.595 0.00969 **

group.L -2.639e-01 1.932e-01 6.616e+02 -1.366 0.17241

region.L -1.765e-01 1.772e-01 1.602e+03 -0.996 0.31921

region.Q 1.542e-01 1.788e-01 1.358e+03 0.863 0.38847

region.C 3.185e-01 1.782e-01 1.119e+03 1.788 0.07411 .

band.L:age.c -4.978e-01 8.055e-02 1.334e+04 -6.180 6.61e-10 ***

band.Q:age.c -7.516e-02 5.242e-02 1.343e+04 -1.434 0.15166

band.L:group.L -3.111e-01 2.637e-01 1.344e+04 -1.180 0.23814

band.Q:group.L -7.548e-02 1.711e-01 1.342e+04 -0.441 0.65919

age.c:group.L -4.172e-02 8.552e-02 6.021e+02 -0.488 0.62584

band.L:region.L -7.336e-01 3.553e-01 1.326e+04 -2.064 0.03899 *

band.Q:region.L -2.707e-01 2.263e-01 1.324e+04 -1.196 0.23160

band.L:region.Q -8.591e-03 3.573e-01 1.334e+04 -0.024 0.98082

band.Q:region.Q 1.547e-01 2.308e-01 1.330e+04 0.671 0.50251

band.L:region.C 8.374e-01 3.521e-01 1.328e+04 2.378 0.01740 *

band.Q:region.C 4.380e-01 2.316e-01 1.327e+04 1.891 0.05862 .

age.c:region.L -1.219e-02 7.430e-02 1.327e+04 -0.164 0.86962

age.c:region.Q 2.427e-02 7.445e-02 1.331e+04 0.326 0.74441

age.c:region.C 1.350e-02 7.421e-02 1.336e+04 0.182 0.85570

group.L:region.L -1.062e-02 2.437e-01 1.327e+04 -0.044 0.96525

group.L:region.Q 8.044e-02 2.454e-01 1.332e+04 0.328 0.74304

group.L:region.C 1.359e-01 2.436e-01 1.329e+04 0.558 0.57704

band.L:age.c:group.L -7.461e-03 1.139e-01 1.334e+04 -0.066 0.94777

band.Q:age.c:group.L -2.867e-02 7.413e-02 1.343e+04 -0.387 0.69895

band.L:age.c:region.L 1.484e-01 1.526e-01 1.326e+04 0.973 0.33064

band.Q:age.c:region.L -9.238e-03 9.863e-02 1.324e+04 -0.094 0.92538

band.L:age.c:region.Q -2.508e-01 1.523e-01 1.333e+04 -1.646 0.09976 .

band.Q:age.c:region.Q 8.275e-03 1.006e-01 1.332e+04 0.082 0.93445

band.L:age.c:region.C -1.643e-01 1.505e-01 1.336e+04 -1.092 0.27503

band.Q:age.c:region.C 6.052e-02 1.006e-01 1.328e+04 0.601 0.54764

band.L:group.L:region.L -1.701e-01 5.024e-01 1.325e+04 -0.339 0.73496

band.Q:group.L:region.L -1.025e-01 3.200e-01 1.324e+04 -0.320 0.74861

band.L:group.L:region.Q 7.135e-01 5.052e-01 1.334e+04 1.412 0.15787

band.Q:group.L:region.Q 1.601e-01 3.263e-01 1.330e+04 0.490 0.62379

band.L:group.L:region.C 7.233e-01 4.980e-01 1.328e+04 1.452 0.14639

band.Q:group.L:region.C 1.390e-01 3.275e-01 1.327e+04 0.425 0.67120

age.c:group.L:region.L 2.634e-02 1.051e-01 1.327e+04 0.251 0.80208

age.c:group.L:region.Q -7.489e-02 1.053e-01 1.331e+04 -0.711 0.47690

age.c:group.L:region.C -1.776e-02 1.049e-01 1.336e+04 -0.169 0.86564

band.L:age.c:group.L:region.L 9.521e-02 2.158e-01 1.325e+04 0.441 0.65903

band.Q:age.c:group.L:region.L 2.195e-02 1.395e-01 1.323e+04 0.157 0.87492

band.L:age.c:group.L:region.Q -2.115e-01 2.154e-01 1.332e+04 -0.982 0.32622

band.Q:age.c:group.L:region.Q -9.653e-02 1.423e-01 1.332e+04 -0.679 0.49745

band.L:age.c:group.L:region.C -2.649e-01 2.128e-01 1.336e+04 -1.245 0.21314

band.Q:age.c:group.L:region.C 4.718e-02 1.423e-01 1.328e+04 0.331 0.74029

---

Signif. codes: 0 ‘***’ 0.001 ‘**’ 0.01 ‘*’ 0.05 ‘.’ 0.1 ‘ ’ 1

**Type III Analysis of Variance Table with Satterthwaite's method**

Sum Sq Mean Sq NumDF DenDF F value Pr(>F)

band 25235.3 12617.6 2 13420.2 2130.6222 < 2.2e-16 ***

age.c 39.9 39.9 1 602.1 6.7333 0.009693 **

group 11.0 11.0 1 661.6 1.8659 0.172414

region 24.5 8.2 3 1177.3 1.3769 0.248270

band:age.c 390.4 195.2 2 13389.9 32.9601 5.257e-15 ***

band:group 17.9 8.9 2 13417.9 1.5077 0.221464

age.c:group 1.4 1.4 1 602.1 0.2380 0.625840

band:region 173.0 28.8 6 13281.9 4.8675 5.631e-05 ***

age.c:region 0.8 0.3 3 13315.2 0.0444 0.987567

group:region 1.9 0.6 3 13300.4 0.1048 0.957320

band:age.c:group 1.4 0.7 2 13387.6 0.1202 0.886710

band:age.c:region 31.1 5.2 6 13286.9 0.8743 0.512723

band:group:region 34.2 5.7 6 13279.2 0.9625 0.448879

age.c:group:region 3.6 1.2 3 13315.0 0.2004 0.896155

band:age.c:group:region 24.9 4.1 6 13284.9 0.7002 0.649488

---

Signif. codes: 0 ‘***’ 0.001 ‘**’ 0.01 ‘*’ 0.05 ‘.’ 0.1 ‘ ’ 1

# Supplemental Table iv. Power at the central frequency of periodic peaks identified by spec_param as a function of linear age, scalp region and group (stroke versus control).

Linear mixed model fit by REML. t-tests use Satterthwaite's method ['lmerModLmerTest']

Formula: log(PW + 1) ~ band * age.c * group * region + (1 | subID) + (1 |Channels)

Data: PEAK_BAND %>% filter(band != "delta")

REML criterion at convergence: -20134.5

Scaled residuals:

Min 1Q Median 3Q Max

-4.2055 -0.6547 -0.0224 0.6137 4.1875

**Random Effects:**

Groups Name Variance Std.Dev.

subID (Intercept) 0.0108369 0.1041

Channels (Intercept) 0.0001513 0.0123

Residual 0.0117283 0.1083

Number of obs: 13465, groups: subID, 295; Channels, 24

**Fixed Effects:**

Estimate Std. Error df t value Pr(>|t|)

(Intercept) 3.252e-01 1.202e-02 3.631e+02 27.046 < 2e-16 ***

band.L -3.817e-02 8.401e-03 1.325e+04 -4.543 5.58e-06 ***

band.Q -1.144e-01 5.454e-03 1.325e+04 -20.978 < 2e-16 ***

age.c -3.816e-03 5.271e-03 3.414e+02 -0.724 0.469677

group.L -2.540e-02 1.656e-02 3.491e+02 -1.534 0.125951

region.L 3.677e-02 9.265e-03 1.557e+02 3.969 0.000110 ***

region.Q 4.134e-02 9.477e-03 1.371e+02 4.362 2.51e-05 ***

region.C -2.036e-02 9.590e-03 1.183e+02 -2.123 0.035820 *

band.L:age.c -3.326e-03 3.641e-03 1.328e+04 -0.913 0.361014

band.Q:age.c 1.774e-02 2.361e-03 1.325e+04 7.514 6.09e-14 ***

band.L:group.L -2.392e-02 1.188e-02 1.324e+04 -2.014 0.044079 *

band.Q:group.L 1.030e-02 7.711e-03 1.325e+04 1.335 0.181764

age.c:group.L 9.137e-07 7.455e-03 3.414e+02 0.000 0.999902

band.L:region.L 1.916e-03 1.585e-02 1.313e+04 0.121 0.903781

band.Q:region.L -1.005e-02 1.009e-02 1.312e+04 -0.996 0.319125

band.L:region.Q -1.256e-02 1.596e-02 1.315e+04 -0.787 0.431313

band.Q:region.Q 3.957e-03 1.030e-02 1.314e+04 0.384 0.700829

band.L:region.C -2.578e-02 1.571e-02 1.313e+04 -1.641 0.100806

band.Q:region.C 3.789e-02 1.033e-02 1.313e+04 3.668 0.000245 ***

age.c:region.L -4.126e-03 3.314e-03 1.313e+04 -1.245 0.213083

age.c:region.Q 2.843e-03 3.323e-03 1.314e+04 0.856 0.392233

age.c:region.C 3.563e-04 3.318e-03 1.316e+04 0.107 0.914485

group.L:region.L 1.061e-02 1.087e-02 1.313e+04 0.976 0.328910

group.L:region.Q 1.535e-02 1.095e-02 1.314e+04 1.401 0.161248

group.L:region.C -2.696e-03 1.087e-02 1.314e+04 -0.248 0.804124

band.L:age.c:group.L -3.911e-03 5.148e-03 1.328e+04 -0.760 0.447477

band.Q:age.c:group.L -6.205e-03 3.339e-03 1.325e+04 -1.858 0.063123 .

band.L:age.c:region.L -2.404e-03 6.803e-03 1.313e+04 -0.353 0.723799

band.Q:age.c:region.L -7.752e-03 4.396e-03 1.312e+04 -1.763 0.077851 .

band.L:age.c:region.Q -6.518e-03 6.802e-03 1.315e+04 -0.958 0.337961

band.Q:age.c:region.Q 6.406e-03 4.492e-03 1.314e+04 1.426 0.153834

band.L:age.c:region.C 8.486e-04 6.726e-03 1.316e+04 0.126 0.899593

band.Q:age.c:region.C 1.732e-03 4.490e-03 1.314e+04 0.386 0.699658

band.L:group.L:region.L 7.108e-03 2.240e-02 1.313e+04 0.317 0.750969

band.Q:group.L:region.L 2.721e-02 1.426e-02 1.312e+04 1.908 0.056407 .

band.L:group.L:region.Q -5.080e-03 2.257e-02 1.315e+04 -0.225 0.821899

band.Q:group.L:region.Q -1.243e-03 1.456e-02 1.314e+04 -0.085 0.931969

band.L:group.L:region.C 2.647e-04 2.222e-02 1.314e+04 0.012 0.990495

band.Q:group.L:region.C -1.035e-02 1.461e-02 1.313e+04 -0.708 0.478769

age.c:group.L:region.L -1.204e-02 4.686e-03 1.313e+04 -2.569 0.010198 *

age.c:group.L:region.Q -2.967e-03 4.699e-03 1.314e+04 -0.631 0.527770

age.c:group.L:region.C -2.459e-03 4.692e-03 1.316e+04 -0.524 0.600203

band.L:age.c:group.L:region.L 2.689e-03 9.620e-03 1.313e+04 0.280 0.779850

band.Q:age.c:group.L:region.L -1.573e-02 6.216e-03 1.312e+04 -2.531 0.011370 *

band.L:age.c:group.L:region.Q 4.862e-04 9.619e-03 1.314e+04 0.051 0.959692

band.Q:age.c:group.L:region.Q 6.758e-04 6.351e-03 1.314e+04 0.106 0.915261

band.L:age.c:group.L:region.C 1.095e-02 9.511e-03 1.316e+04 1.151 0.249807

band.Q:age.c:group.L:region.C 6.779e-03 6.350e-03 1.314e+04 1.068 0.285707

---

Signif. codes: 0 ‘***’ 0.001 ‘**’ 0.01 ‘*’ 0.05 ‘.’ 0.1 ‘ ’ 1

**Type III Analysis of Variance Table with Satterthwaite's method**

Sum Sq Mean Sq NumDF DenDF F value Pr(>F)

band 9.2711 4.6355 2 13250.9 395.2411 < 2.2e-16 ***

age.c 0.0061 0.0061 1 341.4 0.5239 0.469677

group 0.0276 0.0276 1 349.1 2.3530 0.125951

region 0.6558 0.2186 3 125.6 18.6396 4.592e-10 ***

band:age.c 0.7922 0.3961 2 13262.7 33.7711 2.347e-15 ***

band:group 0.0486 0.0243 2 13249.6 2.0730 0.125845

age.c:group 0.0000 0.0000 1 341.4 0.0000 0.999903

band:region 0.2058 0.0343 6 13136.7 2.9250 0.007489 **

age.c:region 0.0279 0.0093 3 13146.9 0.7933 0.497398

group:region 0.0483 0.0161 3 13139.9 1.3725 0.249117

band:age.c:group 0.0858 0.0429 2 13262.1 3.6562 0.025857 *

band:age.c:region 0.1015 0.0169 6 13138.4 1.4425 0.194023

band:group:region 0.0917 0.0153 6 13135.3 1.3027 0.251958

age.c:group:region 0.0834 0.0278 3 13146.8 2.3702 0.068506 .

band:age.c:group:region 0.1429 0.0238 6 13137.8 2.0300 0.058153 .

---

Signif. codes: 0 ‘***’ 0.001 ‘**’ 0.01 ‘*’ 0.05 ‘.’ 0.1 ‘ ’ 1

# Supplemental Table v. Exponent from the power spectrum in people with stroke as a function of scalp region and hemisphere (ipsilesional or contralesional) controlling for age, sex, lesion volume, and time since stroke.

Linear mixed model fit by REML. t-tests use Satterthwaite's method ['lmerModLmerTest']

Formula: Imp_Exp ~ sex + age + days_to_enrollment + lesion_volume + contra *

channel_region + (1 + contra | subID) + (1 | Channels)

Data: STROKE %>% filter(channel_side != "z")

REML criterion at convergence: -69.3

Scaled residuals:

Min 1Q Median 3Q Max

-4.4679 -0.5538 -0.0387 0.5121 4.9123

**Random effects:**

Groups Name Variance Std.Dev. Corr

subID (Intercept) 0.108218 0.32897

contra1 0.001971 0.04439 -0.26

Channels (Intercept) 0.006379 0.07987

Residual 0.039424 0.19855

Number of obs: 1200, groups: subID, 60; Channels, 20

**Fixed effects:**

Estimate Std. Error df t value Pr(>|t|)

(Intercept) 1.416e+00 1.908e-01 5.646e+01 7.418 6.73e-10 ***

sex1 -5.655e-02 4.602e-02 5.498e+01 -1.229 0.224338

age -4.127e-03 3.071e-03 5.504e+01 -1.344 0.184464

days_to_enrollment -2.798e-05 6.905e-05 5.503e+01 -0.405 0.686911

lesion_volume 8.926e-04 1.758e-03 5.518e+01 0.508 0.613653

contra1 -2.079e-02 8.732e-03 7.741e+01 -2.381 0.019715 *

channel_region1 1.284e-01 2.996e-02 1.599e+01 4.286 0.000567 ***

channel_region2 -1.043e-01 3.232e-02 1.599e+01 -3.226 0.005279 **

channel_region3 -5.234e-02 3.657e-02 1.599e+01 -1.431 0.171645

contra1:channel_region1 3.372e-02 9.198e-03 1.061e+03 3.666 0.000258 ***

contra1:channel_region2 -5.411e-03 9.921e-03 1.061e+03 -0.545 0.585559

contra1:channel_region3 -2.241e-02 1.123e-02 1.061e+03 -1.996 0.046196 *

---

Signif. codes: 0 ‘***’ 0.001 ‘**’ 0.01 ‘*’ 0.05 ‘.’ 0.1 ‘ ’ 1

**Type III Analysis of Variance Table with Satterthwaite's method**

Sum Sq Mean Sq NumDF DenDF F value Pr(>F)

sex 0.05954 0.05954 1 54.98 1.5102 0.2243382

age 0.07121 0.07121 1 55.04 1.8063 0.1844636

days_to_enrollment 0.00647 0.00647 1 55.03 0.1642 0.6869110

lesion_volume 0.01016 0.01016 1 55.18 0.2578 0.6136527

contra 0.22353 0.22353 1 77.41 5.6700 0.0197150 *

channel_region 1.15787 0.38596 3 15.99 9.7900 0.0006626 ***

contra:channel_region 0.62011 0.20670 3 1060.77 5.2431 0.0013559 **

---

Signif. codes: 0 ‘***’ 0.001 ‘**’ 0.01 ‘*’ 0.05 ‘.’ 0.1 ‘ ’ 1

# Supplemental Table vi. Number of periodic peaks identified by spec_param in people with stroke as a function of scalp region hemisphere (ipsilesional or contralesional), and frequency band, controlling for age, sex, lesion volume, and time since stroke.

Linear mixed model fit by maximum likelihood . t-tests use Satterthwaite's method [lmerModLmerTest]

Formula: sqrt(peak_count + 1) ~ sex + age + days_to_enrollment + lesion_volume +

band * contra * channel_region + (1 | subID)

Data: PEAK_COUNT %>% filter(band != "delta")

Control: lmerControl(optimizer = "bobyqa", optCtrl = list(maxfun = 5e+05))

AIC BIC logLik -2*log(L) df.resid

-3239.3 -3082.5 1649.6 -3299.3 1345

Scaled residuals:

Min 1Q Median 3Q Max

-1.8318 -0.3199 -0.1035 0.0108 7.6324

**Random Effects:**

Groups Name Variance Std.Dev.

subID (Intercept) 0.0005591 0.02364

Residual 0.0050348 0.07096

Number of obs: 1375, groups: subID, 60

**Fixed Effects:**

Estimate Std. Error df t value Pr(>|t|)

(Intercept) 1.454e+00 1.672e-02 6.070e+01 86.968 < 2e-16 ***

sex.L -9.623e-03 5.679e-03 5.655e+01 -1.695 0.09565 .

age -3.392e-04 2.692e-04 5.879e+01 -1.260 0.21266

days_to_enrollment -9.512e-07 6.161e-06 6.148e+01 -0.154 0.87781

lesion_volume 3.325e-05 1.536e-04 5.896e+01 0.217 0.82933

band.L 1.877e-02 6.277e-03 1.360e+03 2.991 0.00283 **

band.Q 7.710e-03 4.269e-03 1.375e+03 1.806 0.07110 .

contra.L 1.565e-03 4.140e-03 1.340e+03 0.378 0.70549

channel_region.L -1.024e-04 6.241e-03 1.336e+03 -0.016 0.98691

channel_region.Q 1.530e-03 5.861e-03 1.336e+03 0.261 0.79403

channel_region.C -6.333e-04 5.375e-03 1.322e+03 -0.118 0.90623

band.L:contra.L 3.109e-03 8.309e-03 1.339e+03 0.374 0.70836

band.Q:contra.L 2.022e-03 5.801e-03 1.335e+03 0.349 0.72745

band.L:channel_region.L -1.514e-03 1.251e-02 1.335e+03 -0.121 0.90366

band.Q:channel_region.L 1.344e-03 8.757e-03 1.328e+03 0.153 0.87806

band.L:channel_region.Q 6.846e-03 1.178e-02 1.339e+03 0.581 0.56130

band.Q:channel_region.Q 6.751e-03 8.200e-03 1.334e+03 0.823 0.41047

band.L:channel_region.C 5.986e-04 1.084e-02 1.325e+03 0.055 0.95596

band.Q:channel_region.C 5.236e-03 7.494e-03 1.323e+03 0.699 0.48487

contra.L:channel_region.L -1.212e-03 8.784e-03 1.327e+03 -0.138 0.89027

contra.L:channel_region.Q 7.302e-03 8.259e-03 1.334e+03 0.884 0.37679

contra.L:channel_region.C -1.015e-02 7.622e-03 1.327e+03 -1.332 0.18307

band.L:contra.L:channel_region.L 8.794e-04 1.762e-02 1.330e+03 0.050 0.96021

band.Q:contra.L:channel_region.L -1.003e-03 1.238e-02 1.327e+03 -0.081 0.93542

band.L:contra.L:channel_region.Q 1.267e-02 1.661e-02 1.338e+03 0.763 0.44584

band.Q:contra.L:channel_region.Q 7.644e-03 1.156e-02 1.330e+03 0.661 0.50851

band.L:contra.L:channel_region.C -1.840e-02 1.535e-02 1.328e+03 -1.198 0.23098

band.Q:contra.L:channel_region.C -1.044e-02 1.062e-02 1.326e+03 -0.984 0.32539

---

Signif. codes: 0 ‘***’ 0.001 ‘**’ 0.01 ‘*’ 0.05 ‘.’ 0.1 ‘ ’ 1

fit warnings:

Some predictor variables are on very different scales: consider rescaling

**Type III Analysis of Variance Table with Satterthwaite's method**

Sum Sq Mean Sq NumDF DenDF F value Pr(>F)

sex 0.014459 0.014459 1 56.55 2.8717 0.09565 .

age 0.007993 0.007993 1 58.79 1.5875 0.21266

days_to_enrollment 0.000120 0.000120 1 61.48 0.0238 0.87781

lesion_volume 0.000236 0.000236 1 58.96 0.0469 0.82933

band 0.107027 0.053514 2 1370.69 10.6287 2.627e-05 ***

contra 0.000719 0.000719 1 1339.86 0.1429 0.70549

channel_region 0.000676 0.000225 3 1333.32 0.0448 0.98742

band:contra 0.002438 0.001219 2 1332.79 0.2421 0.78503

band:channel_region 0.011957 0.001993 6 1330.49 0.3958 0.88205

contra:channel_region 0.019837 0.006612 3 1330.75 1.3133 0.26849

band:contra:channel_region 0.045051 0.007509 6 1328.75 1.4913 0.17747

---

Signif. codes: 0 ‘***’ 0.001 ‘**’ 0.01 ‘*’ 0.05 ‘.’ 0.1 ‘ ’ 1

# Supplemental Table vii. Central frequency of periodic peaks identified by spec_param in people with stroke as a function of scalp region, hemisphere (ipsilesional or contralesional), and frequency band controlling for age, sex, lesion volume, and time since stroke.

Linear mixed model fit by maximum likelihood . t-tests use Satterthwaite's method [lmerModLmerTest]

Formula: CF ~ sex + age + days_to_enrollment + lesion_volume + band *

contra * channel_region + (1 + contra | subID)

Data: PEAK_BAND %>% filter(band != "delta")

Control: lmerControl(optimizer = "bobyqa", optCtrl = list(maxfun = 5e+05))

AIC BIC logLik -2*log(L) df.resid

5511.2 5680.2 -2723.6 5447.2 1421

Scaled residuals:

Min 1Q Median 3Q Max

-4.1748 -0.4621 -0.0352 0.4685 4.9447

**Random Effects:**

Groups Name Variance Std.Dev. Corr

subID (Intercept) 0.58003 0.7616

contra.L 0.03486 0.1867 -0.18

Residual 2.28228 1.5107

Number of obs: 1453, groups: subID, 60

**Fixed Effects:**

Estimate Std. Error df t value Pr(>|t|)

(Intercept) 1.208e+01 4.837e-01 6.065e+01 24.976 < 2e-16 ***

sex.L -1.198e-01 1.659e-01 5.949e+01 -0.722 0.473097

age -1.813e-02 7.821e-03 5.980e+01 -2.319 0.023847 *

days_to_enrollment 1.757e-04 1.782e-04 6.205e+01 0.986 0.327960

lesion_volume 2.725e-03 4.470e-03 6.024e+01 0.610 0.544398

band.L 6.924e+00 1.349e-01 1.451e+03 51.331 < 2e-16 ***

band.Q 1.855e+00 9.116e-02 1.438e+03 20.353 < 2e-16 ***

contra.L -1.183e-01 9.145e-02 1.822e+02 -1.293 0.197550

channel_region.L -4.734e-01 1.316e-01 1.391e+03 -3.597 0.000333 ***

channel_region.Q -1.763e-01 1.236e-01 1.398e+03 -1.426 0.154121

channel_region.C -8.251e-02 1.135e-01 1.378e+03 -0.727 0.467336

band.L:contra.L -1.402e-01 1.782e-01 9.343e+02 -0.786 0.431809

band.Q:contra.L -1.840e-01 1.235e-01 1.201e+03 -1.490 0.136572

band.L:channel_region.L -8.583e-01 2.634e-01 1.397e+03 -3.258 0.001147 **

band.Q:channel_region.L -7.004e-01 1.845e-01 1.385e+03 -3.797 0.000153 ***

band.L:channel_region.Q 5.033e-01 2.493e-01 1.403e+03 2.018 0.043739 *

band.Q:channel_region.Q -9.627e-02 1.728e-01 1.392e+03 -0.557 0.577575

band.L:channel_region.C -2.400e-02 2.292e-01 1.382e+03 -0.105 0.916605

band.Q:channel_region.C -1.259e-01 1.578e-01 1.375e+03 -0.798 0.425210

contra.L:channel_region.L 7.664e-02 1.852e-01 1.398e+03 0.414 0.679102

contra.L:channel_region.Q -6.673e-02 1.745e-01 1.403e+03 -0.382 0.702185

contra.L:channel_region.C 4.063e-02 1.609e-01 1.372e+03 0.252 0.800713

band.L:contra.L:channel_region.L 3.143e-01 3.715e-01 1.400e+03 0.846 0.397682

band.Q:contra.L:channel_region.L 1.310e-01 2.608e-01 1.387e+03 0.502 0.615478

band.L:contra.L:channel_region.Q 1.403e-01 3.513e-01 1.406e+03 0.399 0.689781

band.Q:contra.L:channel_region.Q -1.814e-01 2.440e-01 1.397e+03 -0.743 0.457334

band.L:contra.L:channel_region.C -2.558e-01 3.247e-01 1.377e+03 -0.788 0.431009

band.Q:contra.L:channel_region.C 9.430e-02 2.236e-01 1.372e+03 0.422 0.673236

---

Signif. codes: 0 ‘***’ 0.001 ‘**’ 0.01 ‘*’ 0.05 ‘.’ 0.1 ‘ ’ 1

fit warnings:

Some predictor variables are on very different scales: consider rescaling

**Type III Analysis of Variance Table with Satterthwaite's method**

Sum Sq Mean Sq NumDF DenDF F value Pr(>F)

sex 1.2 1.2 1 59.49 0.5213 0.473097

age 12.3 12.3 1 59.80 5.3764 0.023847 *

days_to_enrollment 2.2 2.2 1 62.05 0.9722 0.327960

lesion_volume 0.8 0.8 1 60.24 0.3717 0.544398

band 11843.1 5921.6 2 1439.40 2594.5768 < 2.2e-16 ***

contra 3.8 3.8 1 182.19 1.6726 0.197550

channel_region 30.2 10.1 3 1392.62 4.4062 0.004301 **

band:contra 12.0 6.0 2 1036.74 2.6285 0.072664 .

band:channel_region 152.0 25.3 6 1386.91 11.1015 4.016e-12 ***

contra:channel_region 1.7 0.6 3 1393.16 0.2537 0.858739

band:contra:channel_region 8.6 1.4 6 1386.70 0.6300 0.706413

---

Signif. codes: 0 ‘***’ 0.001 ‘**’ 0.01 ‘*’ 0.05 ‘.’ 0.1 ‘ ’ 1

# Supplemental Table viii. Power at the central frequency of periodic peaks identified by spec_param in people with stroke as a function of scalp region, hemisphere (ipsilesional or contralesional), and frequency band controlling for age, sex, lesion volume, and time since stroke.

Linear mixed model fit by maximum likelihood . t-tests use Satterthwaite's method [

lmerModLmerTest]

Formula: log(PW + 1) ~ sex + age + days_to_enrollment + lesion_volume +

band * contra * channel_region + (1 + contra | subID) + (1 |Channels)

Data: PEAK_BAND %>% filter(band != "delta")

Control: lmerControl(optimizer = "bobyqa", optCtrl = list(maxfun = 5e+05))

AIC BIC logLik deviance df.resid

-2477.9 -2303.6 1272.0 -2543.9 1420

Scaled residuals:

Min 1Q Median 3Q Max

-3.4435 -0.6463 0.0123 0.6625 3.6539

**Random effects:**

Groups Name Variance Std.Dev. Corr

subID (Intercept) 0.0090781 0.09528

contra.L 0.0012258 0.03501 -0.07

Channels (Intercept) 0.0002534 0.01592

Residual 0.0084270 0.09180

Number of obs: 1453, groups: subID, 60; Channels, 20

**Fixed effects:**

Estimate Std. Error df t value Pr(>|t|)

(Intercept) 3.904e-01 5.674e-02 5.910e+01 6.881 4.26e-09 ***

sex.L 1.609e-02 1.946e-02 5.870e+01 0.827 0.411758

age -1.192e-03 9.168e-04 5.847e+01 -1.301 0.198525

days_to_enrollment 1.016e-05 2.072e-05 5.949e+01 0.490 0.625622

lesion_volume -1.097e-03 5.241e-04 5.840e+01 -2.094 0.040602 *

band.L -6.043e-02 8.423e-03 1.387e+03 -7.175 1.18e-12 ***

band.Q -7.855e-02 5.659e-03 1.376e+03 -13.880 < 2e-16 ***

contra.L 1.220e-02 7.213e-03 1.248e+02 1.691 0.093333 .

channel_region.L 3.040e-02 1.193e-02 2.771e+01 2.548 0.016677 *

channel_region.Q -5.495e-02 1.114e-02 2.840e+01 -4.932 3.23e-05 ***

channel_region.C 8.608e-04 1.019e-02 2.822e+01 0.085 0.933255

band.L:contra.L -1.263e-02 1.149e-02 1.305e+03 -1.099 0.271889

band.Q:contra.L -3.897e-03 7.818e-03 1.360e+03 -0.498 0.618265

band.L:channel_region.L 2.151e-02 1.617e-02 1.350e+03 1.330 0.183713

band.Q:channel_region.L -4.398e-02 1.130e-02 1.344e+03 -3.891 0.000105 ***

band.L:channel_region.Q 2.774e-02 1.534e-02 1.355e+03 1.808 0.070851 .

band.Q:channel_region.Q -1.342e-02 1.060e-02 1.348e+03 -1.265 0.206079

band.L:channel_region.C 9.597e-04 1.405e-02 1.345e+03 0.068 0.945550

band.Q:channel_region.C -5.135e-03 9.657e-03 1.338e+03 -0.532 0.594999

contra.L:channel_region.L 2.264e-02 1.144e-02 1.366e+03 1.979 0.048058 *

contra.L:channel_region.Q -8.830e-03 1.079e-02 1.367e+03 -0.818 0.413297

contra.L:channel_region.C 1.263e-02 9.887e-03 1.344e+03 1.278 0.201615

band.L:contra.L:channel_region.L -5.514e-02 2.289e-02 1.359e+03 -2.409 0.016135 *

band.Q:contra.L:channel_region.L 7.460e-03 1.597e-02 1.342e+03 0.467 0.640444

band.L:contra.L:channel_region.Q -3.756e-03 2.173e-02 1.367e+03 -0.173 0.862773

band.Q:contra.L:channel_region.Q 2.388e-02 1.497e-02 1.348e+03 1.595 0.110904

band.L:contra.L:channel_region.C -1.136e-02 1.994e-02 1.346e+03 -0.570 0.569104

band.Q:contra.L:channel_region.C 1.304e-02 1.366e-02 1.335e+03 0.954 0.340026

---

Signif. codes: 0 ‘***’ 0.001 ‘**’ 0.01 ‘*’ 0.05 ‘.’ 0.1 ‘ ’ 1

**Type III Analysis of Variance Table with Satterthwaite's method**

Sum Sq Mean Sq NumDF DenDF F value Pr(>F)

sex 0.0058 0.00576 1 58.70 0.6834 0.411758

age 0.0143 0.01425 1 58.47 1.6914 0.198525

days_to_enrollment 0.0020 0.00203 1 59.49 0.2405 0.625622

lesion_volume 0.0370 0.03695 1 58.40 4.3852 0.040602 *

band 3.6879 1.84396 2 1376.62 218.8146 < 2.2e-16 ***

contra 0.0241 0.02410 1 124.83 2.8595 0.093333 .

channel_region 0.4577 0.15255 3 27.68 18.1030 1.06e-06 ***

band:contra 0.0219 0.01096 2 1328.26 1.3010 0.272604

band:channel_region 0.1499 0.02498 6 1345.67 2.9640 0.007044 **

contra:channel_region 0.0879 0.02931 3 1364.19 3.4784 0.015459 *

band:contra:channel_region 0.0991 0.01651 6 1350.13 1.9594 0.068446 .

---

Signif. codes: 0 ‘***’ 0.001 ‘**’ 0.01 ‘*’ 0.05 ‘.’ 0.1 ‘ ’ 1

# Supplemental Table ix. Associations between the Box and Block Test (BBT) and the exponent of the power spectrum in people with stroke as a function of scalp region, hemisphere (ipsilesional or contralesional), and frequency band controlling for age, sex, and time since stroke.

Linear mixed model fit by maximum likelihood . t-tests use Satterthwaite's method ['lmerModLmerTest']

Formula: Imp_Exp ~ sex + days_to_enrollment.c + lesion_volume.c + bbt_affected.c *

age.c * channel_region * contra + (1 + contra | subID) + (1 | Channels)

Data: STROKE

Control: lmerControl(optimizer = "bobyqa", optCtrl = list(maxfun = 5e+05))

AIC BIC logLik deviance df.resid

-71.2 113.1 75.6 -151.2 700

Scaled residuals:

Min 1Q Median 3Q Max

-4.5725 -0.5346 -0.0850 0.5249 5.0718

**Random effects:**

Groups Name Variance Std.Dev. Corr

subID (Intercept) 0.089217 0.29869

contra.L 0.002255 0.04749 0.17

Channels (Intercept) 0.004423 0.06650

Residual 0.036739 0.19167

Number of obs: 740, groups: subID, 37; Channels, 20

**Fixed effects:**

Estimate Std. Error df t value Pr(>|t|)

(Intercept) 1.151e+00 5.764e-02 4.367e+01 19.970 < 2e-16 ***

sex.L 1.127e-01 7.887e-02 3.699e+01 1.430 0.161246

days_to_enrollment.c -8.397e-05 8.297e-05 3.698e+01 -1.012 0.318090

lesion_volume.c -1.093e-03 2.220e-03 3.698e+01 -0.492 0.625316

bbt_affected.c 1.112e-02 3.953e-03 3.745e+01 2.813 0.007781 **

age.c -5.568e-03 3.803e-03 3.742e+01 -1.464 0.151485

channel_region.L -4.909e-02 4.063e-02 1.947e+01 -1.208 0.241453

channel_region.Q 1.571e-01 3.772e-02 1.947e+01 4.164 0.000504 ***

channel_region.C -5.838e-02 3.458e-02 1.947e+01 -1.688 0.107294

contra.L 3.511e-02 1.426e-02 5.189e+01 2.463 0.017149 *

bbt_affected.c:age.c 1.048e-04 2.281e-04 3.742e+01 0.460 0.648477

bbt_affected.c:channel_region.L 7.540e-04 1.194e-03 6.476e+02 0.631 0.528074

bbt_affected.c:channel_region.Q 1.917e-03 1.109e-03 6.476e+02 1.729 0.084293 .

bbt_affected.c:channel_region.C -2.736e-04 1.016e-03 6.476e+02 -0.269 0.787839

age.c:channel_region.L 4.769e-03 1.259e-03 6.476e+02 3.788 0.000166 ***

age.c:channel_region.Q 4.591e-04 1.169e-03 6.476e+02 0.393 0.694648

age.c:channel_region.C -1.338e-03 1.071e-03 6.476e+02 -1.249 0.212294

bbt_affected.c:contra.L -2.050e-04 9.973e-04 5.437e+01 -0.206 0.837879

age.c:contra.L 7.005e-04 1.012e-03 5.195e+01 0.692 0.492083

channel_region.L:contra.L 4.811e-02 2.534e-02 6.541e+02 1.898 0.058111 .

channel_region.Q:contra.L -3.217e-02 2.353e-02 6.541e+02 -1.367 0.172070

channel_region.C:contra.L 2.193e-03 2.157e-02 6.541e+02 0.102 0.919054

bbt_affected.c:age.c:channel_region.L -1.030e-04 7.534e-05 6.476e+02 -1.367 0.172105

bbt_affected.c:age.c:channel_region.Q 1.712e-04 6.995e-05 6.476e+02 2.448 0.014643 *

bbt_affected.c:age.c:channel_region.C -3.301e-05 6.411e-05 6.476e+02 -0.515 0.606771

bbt_affected.c:age.c:contra.L -6.271e-05 6.242e-05 5.388e+01 -1.005 0.319518

bbt_affected.c:channel_region.L:contra.L 1.188e-03 1.781e-03 6.670e+02 0.667 0.505007

bbt_affected.c:channel_region.Q:contra.L -2.915e-03 1.653e-03 6.670e+02 -1.763 0.078344 .

bbt_affected.c:channel_region.C:contra.L -1.447e-03 1.515e-03 6.670e+02 -0.955 0.340042

age.c:channel_region.L:contra.L 1.425e-03 1.800e-03 6.546e+02 0.792 0.428743

age.c:channel_region.Q:contra.L 2.666e-03 1.671e-03 6.546e+02 1.595 0.111173

age.c:channel_region.C:contra.L -7.339e-04 1.532e-03 6.546e+02 -0.479 0.632034

bbt_affected.c:age.c:channel_region.L:contra.L -1.206e-06 1.113e-04 6.663e+02 -0.011 0.991361

bbt_affected.c:age.c:channel_region.Q:contra.L -1.323e-04 1.034e-04 6.663e+02 -1.280 0.201148

bbt_affected.c:age.c:channel_region.C:contra.L -6.657e-06 9.475e-05 6.663e+02 -0.070 0.944008

---

Signif. codes: 0 ‘***’ 0.001 ‘**’ 0.01 ‘*’ 0.05 ‘.’ 0.1 ‘ ’ 1

**Type III Analysis of Variance Table with Satterthwaite's method**

Sum Sq Mean Sq NumDF DenDF F value Pr(>F)

sex 0.07508 0.07508 1 36.99 2.0435 0.1612460

days_to_enrollment.c 0.03763 0.03763 1 36.98 1.0243 0.3180900

lesion_volume.c 0.00891 0.00891 1 36.98 0.2425 0.6253160

bbt_affected.c 0.29062 0.29062 1 37.45 7.9106 0.0077811 **

age.c 0.07877 0.07877 1 37.42 2.1440 0.1514846

channel_region 1.35954 0.45318 3 19.47 12.3352 9.534e-05 ***

contra 0.22280 0.22280 1 51.89 6.0646 0.0171489 *

bbt_affected.c:age.c 0.00776 0.00776 1 37.42 0.2112 0.6484766

bbt_affected.c:channel_region 0.13432 0.04477 3 647.59 1.2187 0.3020013

age.c:channel_region 0.66354 0.22118 3 647.59 6.0204 0.0004774 ***

bbt_affected.c:contra 0.00155 0.00155 1 54.37 0.0423 0.8378793

age.c:contra 0.01759 0.01759 1 51.95 0.4787 0.4920830

channel_region:contra 0.34871 0.11624 3 654.06 3.1639 0.0240820 *

bbt_affected.c:age.c:channel_region 0.53321 0.17774 3 647.59 4.8378 0.0024490 **

bbt_affected.c:age.c:contra 0.03709 0.03709 1 53.88 1.0095 0.3195178

bbt_affected.c:channel_region:contra 0.20883 0.06961 3 667.02 1.8947 0.1291390

age.c:channel_region:contra 0.13019 0.04340 3 654.63 1.1812 0.3160275

bbt_affected.c:age.c:channel_region:contra 0.07590 0.02530 3 666.30 0.6886 0.5591791

---

Signif. codes: 0 ‘***’ 0.001 ‘**’ 0.01 ‘*’ 0.05 ‘.’ 0.1 ‘ ’ 1
